# Positive interactions of major-effect QTLs with genetic background that enhances rice yield under drought

**DOI:** 10.1038/s41598-018-20116-7

**Published:** 2018-01-26

**Authors:** Nitika Sandhu, Shalabh Dixit, B. P. Mallikarjuna Swamy, Prashant Vikram, Challa Venkateshwarlu, Margaret Catolos, Arvind Kumar

**Affiliations:** 1 International Rice Research Institute, DAPO BOX 7777 Metro Manila, Philippines; 2 International Maize and Wheat Improvement Center (CIMMYT), Mexico, Mexico

## Abstract

To improve the grain yield of the lowland-adapted popular rice variety Samba Mahsuri under reproductive-stage drought (RS) and to understand the interactions between drought QTLs, two mapping populations were developed using marker-assisted selection (MAS) and marker-assisted recurrent selection (MARS). The mean grain yield of pyramided lines (PLs) with *qDTY*_*2.2*_ + *qDTY*_*4.1*_ in MAS is significantly higher under RS and irrigated control than lines with single QTLs. Among MARS PLs, lines with four *qDTYs* (*qDTY*_*1.1*_ + *qDTY*_*2.1*_ + *qDTY*_*3.1*_ + *qDTY*_*11.1*_) and two QTLs (*qDTY*_*1.1*_ + *qDTY*_*11.1*_) yielded higher than PLs with other *qDTY* combinations. The selected PLs showed a yield advantage of 0.3–2.0 t ha^−1^ under RS. An allelic profile of MAS PLs having same *qDTY* combination but with different yields under drought was studied. Hierarchical clustering grouped together the selected lines with high yield under drought. Epistasis test showed the interaction of *qDTY*_*4.1*_ and *qDTY*_*9.1*_ loci with *qDTY*_*7.1*_ significantly increased yield under drought and all the lines with higher yield under drought possessed the conserved region of *qDTY*_*7.1*_ on chromosome 7. The positive interactions among QTLs, effectiveness of QTLs in different backgrounds, introgression of *DTY* QTLs together with resistance to biotic stresses shall help enhance grain yield under RS.

## Introduction

The increasing population, declining water availability with increasing demand, climate change and abiotic stresses are serious threats to world food security. Global rice consumption is reported to increase 8.8% from 2011 to 2020 and about 32.7% by 2050^1^. Drought is a recurring global, climatically induced calamity that affects rice production in arid, semi-arid, and humid areas as well. Tackling drought can provide excellent returns to rice research and development investments. In India, severe drought in 2002 and 2009 caused a 20% and 16% reduction in food grain, respectively, leading to higher prices and food security concerns^2^. In 2004, severe drought affected the crop on more than 2 million ha in Southeast Asia^2^. An effective breeding approach to develop drought-tolerant rice varieties could lead to food security under situations of accelerating food demand, depleting resources, and predicated climatic variability^3^.

Various strategies such as conventional hybridization and selection procedures, ideotype breeding, heterosis breeding, wide hybridization, molecular breeding^3^, and new genomics tools and technologies can be used to increase yield potential. The marker-assisted breeding program at IRRI has led to the identification of 12 major drought yield QTLs (*qDTY*_*1.1*_, *qDTY*_*2.1*_, *qDTY*_*2.2*_, *qDTY*_*2.3*_, *qDTY*_*3*__.__*1*_, *qDTY*_*3.2*_, *qDTY*_*4.1*_, *qDTY*_*6.1*_, *qDTY*_*6.2*_, *qDTY*_*9.1*_*, qDTY*_*10.1*_ and *qDTY*_*12*__.__*1*_) in the background of high yielding varieties-IR64, MTU1010, Swarna, Sabitri, TDK1, and Vandana^4–12^. Seven *DTY* QTLs-*qDTY*_*1*__.__*1*_^6^, *qDTY*_*2.2*_^11,13^, *qDTY*_*3*__.__*1*_^5,6^*, qDTY*_*3.2*_^9^*, qDTY*_*4*__.__*1*_^13^, *qDTY*_*6*__.__*1*_^6^, and *qDTY*_*12*__.__*1*_^4,11^ have shown large effects across two or more genetic backgrounds and under both transplanted lowland and direct seeded upland environments.

The use of different genotyping approaches^6–14^, the identification of traits of interest, and major genetic regions associated with the targeted traits have opened new opportunities to successfully introgress/pyramid genes/QTLs in different genetic backgrounds using marker-assisted backcrossing^4–9,15–17^ and marker-assisted recurrent selection^18^. To achieve the desirable phenotypic level of variation for a quantitative trait, pyramiding QTLs may be an effective approach^19^. The effect of identified genetic loci in a pyramiding program is not always as expected because of the complexity of gene networks, epistasis, pleiotropy, and linkage interactions among/between genetic regions as well as haplotype groups with the environment^20,21^. QTL pyramiding will not only help in understanding the interactions among genetic loci but also improve the efficiency of marker-assisted selection for desirable loci in rice breeding programs. Under severe drought stress, a grain yield advantage of 0.8–1.0 t ha^−1^ was reported in a QTL introgression program involving popular high-yielding variety IR64 through the introgression of two QTLs (*qDTY*_*2.2*_ and *qDTY*_*4.1*_)^13^. Positive QTL interactions with a significant increase in grain yield under drought stress without a yield penalty under control conditions were reported by Dixit *et al*.^22^ and Shamsudin *et al*.^23^.

Marker-assisted selection can generate an improved version of an existing elite genotype as it involves the transfer of favorable alleles from a donor to a recipient parent. Breeding approaches such as marker-assisted recurrent selection (MARS) involve taking advantage of elite alleles coming from two or more parental lines can be a viable alternative. MARS, a practice of improvement of low heritability traits^24,25^, involves the consideration of selection intensity to increase the frequency of favorable superior genes^21^ and genetic drift to maintain diversity to carry on further improvement^26^. MARS has the potential to expand the gene pool of present cultivars and to expedite the development of new varieties. MARS involves selecting genotypes based on their favorable allele combinations and intermating them to produce the next generation^27–30^. Repeated intermating of heterozygous populations helps in successfully maintaining and escalating the genetic gain and variability^31–34^ resulting from the optimum complement from both parents. Validation of the effects of QTLs that showed large and consistent effects in one background will promote their extensive use across different genetic backgrounds for increasing yield under drought. In the present study, two mapping populations were developed following marker-assisted breeding and marker-assisted recurrent selection using rice variety Samba Mahsuri as the recipient parent. The objectives of the study were to (1) evaluate the effects of *qDTY*_*2.2*_ and *qDTY*_*4.1*_ in a Samba Mahsuri background using the MAB approach, which had earlier shown effects in an IR64 background; (2) understand the interactions of drought grain yield QTLs *qDTY*_*1*__.__*1*_, *qDTY*_*2*__.__*1*_, *qDTY*_*3*__.__*1*_, and *qDTY*_*11*__.__*1*_ in a Samba Mahsuri background using the MARS breeding approach; and (3) develop high-yielding blast-and drought-tolerant Samba Mahsuri PLs for cultivation by farmers.

## Results

### Marker-assisted introgression for *qDTY*_*2.2*_ + *qDTY*_*4.1*_ in Samba Mahsuri background

The mapping population was developed from crosses of drought-tolerant donor IR 87728-75-B-B possessing *qDTY*_*2.2*_ and *qDTY*_*4.1*_ with drought-susceptible Samba Mahsuri in DS 2011 (DS: dry season) to introgress and pyramid QTLs for grain yield under drought. The earlier reported markers for *qDTY*_*2.2*_ (RM236, RM279, RM109) and *qDTY*_*4.1*_ (RM335, RM551, RM518) in an IR64 background^13^ were used to genotype the population. Foreground and recombinant genotyping was used to identify true F_1_ in WS 2011 (WS: wet season). The grain type of the selected marker assisted lines is provided in Supplementary Fig. S1. The complete scheme for the development of Samba Mahsuri pyramided lines and number of plants selected based on genotyping, phenotyping involving plant type, visual yield under drought stress and non-stress, and bacterial blight resistance is provided in Supplementary Fig. S2.

### Marker-assisted recurrent selection for *qDTY*_*1.1*_, *qDTY*_*2.1*_, *qDTY*_*3.1*_, and *qDTY*_*11.1*_ in Samba Mahsuri background

A mapping population was developed from crosses of drought-tolerant donor IR55419-04 possessing *qDTY*_*1.1*_*, qDTY*_*2.1*_, *qDTY*_*3.1*_, and *qDTY*_*11.1*_ with drought-susceptible high-yielding Samba Mahsuri in DS 2008 followed by one backcross in WS 2008 to introgress and pyramid QTLs for grain yield under drought. The lines were advanced in DS 2009, WS 2009, and DS 2010. A total of 500 BC_1_F_4_ lines were selected based on plant type and Samba Mahsuri grain type in WS 2010 and advanced in DS 2011. A total of 686 panicles with Samba Mahsuri grain type were selected and advanced in WS 2011. The lines with different QTL combinations and the highest phenotypic similarity to the recipient parent and good grain yield under drought stress and non-stress were selected in the BC_1_F_6_ population and intercrossed in DS 2012. In WS 2012, 15641 F_1_ seeds were produced. In each intercross F_2_ generation, selection was done based on grain type similar to the recipient type, blast resistance (*Magnaporthe oryzae*) and bacterial blight (*Xanthomonas oryzae* pv*. Oryzae*) resistance, and foreground and recombinant selection were practiced using the peak and other foreground markers RM212 and RM486 (chromosome 1), RM525 and RM221 (chromosome 2), RM16 and RM520 (chromosome 3), and RM287 (chromosome 11) to select plants segregating for the respective introgressed drought QTLs in DS 2013. Plants fixed for different combinations of QTLs, grain type (Supplementary Fig. S1), plant type and visual yield were selected in each generation and advanced to F_8_ generations. The scheme for the development of Samba Mahsuri PLs using partial marker-assisted recurrent selection is provided in Supplementary Fig. S3.

### Phenotypic evaluation of populations

The average days to flowering (DTF) of PLs in the marker-assisted backcross experiment varied from 79 to 88 days in the NS trials and from 82 to 98 days in the reproductive-stage (RS) drought stress trials (Table 1). At maturity, plant height (PHT) varied from 62 to 71 cm in the reproductive-stage drought stress trials and from 81 to 105 cm in the NS trials (Table 1). Plant height and DTF were severely affected by drought stress as reflected by the reduction of 19 to 34 cm in height and delayed flowering by 3 to 10 days. Grain yield (GY) varied from 114 to 1366 kg ha^−1^ in the RS drought stress experiments and from 2376 to 8133 kg ha^−1^ in the NS experiments (Table 1). The grain yield reduction of 83% to 95% under drought stress compared with NS (control) indicated the severity of reproductive-stage drought stress faced by PLs. In the marker-assisted recurrent selection experiment, the average DTF of PLs varied from 80 to 97 days in the NS trials and from 85 to 116 days in the RS trials (Table 1). Plant height varied from 99 to 113 cm in the NS trials and from 73 to 85 cm in the RS drought stress trials (Table 1). Plant height decreased by 26 to 28 cm and flowering was delayed by 5 to 19 days. Grain yield ranged from 126 to 1751 kg ha^−1^ in the RS experiments and from 3131 to 5824 kg ha^−1^ in the NS experiments (Table 1). The PLs IR 102818-10-266-3-2-2-6, IR 102818-10-276-1-2-2-9, IR 102818-10-227-1-2-1-9, IR 102818-10-227-1-2-1-6, IR 99734:1-33-69-1-39-6, IR 99734:1-33-69-1-12-8, and IR 99734:1-33-69-1-12-9 showed resistance to blast (*Magnaporthe oryzae*). The PLs IR 99734:1-33-304-1-5-10 and IR 99734:1-33-304-1-5-8 showed resistance to bacterial blight (*Xanthomonas oryzae* pv*. Oryzae*) and mild resistance to blast (*Magnaporthe oryzae*).

**Table 1:** Means for agronomic traits of pyramided lines (PLs) compared with recipient parent under lowland reproductive-stage drought stress (RS) and non-stress (NS) conditions using marker-assisted selection and marker-assisted recurrent selection approaches.

Experiment	Location	Population size	Experimental design	Season	Environment	DTF (days)		PHT (cm)		GY (kg ha^−1^)	
Marker-assisted selection						PLs	Samba Mahsuri	PLs	Samba Mahsuri	PLs	Samba Mahsuri
	Philippines	45	Augmented RCBD	DS 2013	NS	83 ± 1	89	81 ± 1	80	2376 ± 118	2486
	Philippines	45	Augmented RCBD	DS 2013	RS	85 ± 2	—	69 ± 3	—	1366 ± 485	0
	Philippines	644	Augmented RCBD	DS 2014	NS	84 ± 4	102	91 ± 6	87	8133 ± 736	9753
	Philippines	644	161 × 4 AL	DS 2014	RS	92 ± 4	—	62 ± 5	—	850 ± 556	0
	Philippines	70	10 × 7 AL	WS 2014	NS	88 ± 2	100	100 ± 3	95	3301 ± 488	2137
	Philippines	148	Augmented RCBD	DS 2015	NS	88 ± 1	100	86 ± 7	85	6144 ± 1208	5325
	Philippines	70	7 × 10 AL	WS 2015	NS	87 ± 1	105	105 ± 4	101	4180 ± 569	4044
	Philippines	70	7 × 10 AL	WS 2015	RS	98 ± 7	130	71 ± 6	74	114 ± 126	48
	Philippines	18	3 × 6 AL	DS 2016	NS	79 ± 1	93	89 ± 1	77	4575 ± 184	4951
	Philippines	18	3 × 6 AL	DS 2016	RS	82 ± 3	—	66 ± 2	—	222 ± 110	0
	Hyderabad	70	10 × 7 AL	WS 2014	NS	88 ± 1	101	97 ± 4	86	6285 ± 557	4762
	Hyderabad	70	10 × 7 AL	WS 2014	RS	96 ± 1	111	61 ± 2	53	322 ± 35	0
	Hyderabad	28	7 × 4 AL	DS 2015	NS	86 ± 3	112	89 ± 3	79	5858 ± 880	7403
	Hyderabad	25	RCBD	WS 2015	RS	97 ± 1	—	56 ± 1	—	658 ± 59	0
Marker-assisted recurrent selection	Philippines	260	Augmented RCBD	DS 2014	NS	88 ± 6	102	105 ± 6	84	4389 ± 1186	3456
	Philippines	260	52 × 5 AL	DS 2014	RS	92 ± 3	112	79 ± 6	54	1751 ± 375	103
	Philippines	58	Augmented RCBD	WS 2014	NS	93 ± 3	103	109 ± 5	95	3131 ± 1126	1590
	Philippines	152	Augmented RCBD	DS 2015	NS	91 ± 15	99	99 ± 8	68	5824 ± 1315	3137
	Philippines	60	6 × 10 AL	WS 2015	NS	97 ± 2	108	116 ± 8	87	3186 ± 688	1124
	Philippines	60	6 × 10 AL	WS 2015	RS	116 ± 4	131	85 ± 6	74	126 ± 101	15
	Philippines	30	3 × 10 AL	DS 2016	NS	80 ± 2	76	100 ± 3	81	4370 ± 388	4203
	Philippines	30	3 × 10 AL	DS 2016	RS	88 ± 4	—	73 ± 6	—	156 ± 73	0

NS: Non stress, RS: Reproductive stage drought stress, DTF: Days to 50% flowering, PHT: Plant height, GY: Grain Yield, DS: Dry season, WS: Wet season, Samba Mahsuri: recipient parent (no QTL).

### QTL class analysis of PLs

The mean grain yield of PLs with single and different QTL combinations for marker-assisted backcross (QTL class – A, B, and C) and marker-assisted recurrent selection experiments (QTL class – A, B, C, D, E, F, G, H, I, J, K, and L) together with the check (CH) and parents (X, P1, P2) is shown in Table 2, Supplementary Fig. S4 (A,B) and Table 3, Supplementary Fig. S4 (C,D) respectively.

**Table 2:** QTL class analysis of grain yield (kg ha^−1^) under reproductive-stage drought stress (RS) and irrigated non-stress (NS) control conditions in marker-assisted backcross experiment conducted at IRRI, Philippines, and Hyderabad, India.

Class	QTL	WS 2014 Phil	WS 2015 Phil		DS 2016 Phil		WS 2014 HYD		DS 2015 HYD	WS 2015 HYD	
		*NS*	*NS*	*RS*	*NS*	*RS*	*NS*	*RS*	*NS*	*NS*	*RS*
A	*q* *DTY*_*2.2*_	3404.73b	3327.46a	44.36a	—	—	6308.66b	388.25b	5334.68a	7437.65b	678.10a
B	*q* *DTY*_*4.1*_	3339.73b	4726.67c	183.84b	5642.76b	33.66a	6433.50b	335.69b	6400.64cb	7414.79b	638.39a
C	*qDTY*_*2.2*_ + *qDTY*_*4.1*_	3270.35b	4161.38 b	110.16ba	4299.29a	215.65b	6305.25b	322.03b	5748.88ab	7704.38b	515.62a
CH	IR87707-445-B-B-B:6	3919.44c	6272.56d	192.24b	5336.93b	871.84c	7270.28c	395.66b	6273.2cb	9821.59c	1000.34a
X	Samba Mahsuri	2136.95a	4044.49b	48.65a	4951.25b	0a	4761.60a	0a	7402.84c	3347.67a	—
F-value		11.18	43.03	2.12	19.98	62.66	21.61	1.64	4.33	43.42	1.89
p-value		<0.0001	<0.0001	0.019	<0.0001	<0.0001	<0.0001	0.1858	0.0104	<0.0001	0.2323

Means followed by the same letter (within a column) are not significantly different (F-test), NS: non-stress, RS: reproductive-stage drought stress, HYD: Hyderabad, Phil: Philippines, CH: drought-tolerant check, X: recipient parent (no QTL).

**Table 3:** QTL class analysis of grain yield (kg ha^−1^) under reproductive-stage drought stress (RS) and irrigated non-stress (NS) control conditions in marker-assisted recurrent selection experiment conducted at IRRI, Philippines.

Class	QTL	DS 2014		WS 2014	DS 2015		WS 2015		DS 2016	
		NS	RS	NS	NS	RS	NS	RS	NS	RS
A	*qDTY*_*1.1*_ + *qDTY*_*2.1*_ + *qDTY*_*3.1*_* + qDTY*_*11.1*_ + BR	4166.17ced	2032.96 f	2604.11cb	5602.44 cd	599.60bd	4547.82d	285.44dc	3560.22ab	541.46c
B	*qDTY*_*1.1*_ + *qDTY*_*2.1*_ + *qDTY*_*3.1*_ + *qDTY*_*11.1*_	4653.41e	1847.47de	3070.29eb	7244.03 g	562.69bc	—	—	—	—
C	*qDTY*_*1.1*_ + *qDTY*_*2.1*_ + *qDTY*_*3.1*_	4507.37e	1796.12e	3198.70eb	5884.82cf	668.018dc	—	—	—	—
D	*qDTY*_*1.1*_ + *qDTY*_*2.1*_ + *qDTY*_*3.1*_ + BR	4405.75eb	1962.99fe	4777.08e	6785.81gdfe	556.69bd	—	—	—	—
E	*qDTY*_*1.1*_ + *qDTY*_*2.1*_ + *qDTY*_*11.1*_	4971.54cedf	1951.36fe	—	6920.59ge	497.21bc	—	—	—	—
F	*qDTY*_*1.1*_ + *qDTY*_*2.1*_ + *qDTY*_*11.1*_ + BR	4315.05ced	1826.22e	3474.91ecd	7321.31 g	670.09bd	3396.08bc	66.79a	4688.11d	90.73a
G	*qDTY*_*2.1*_ + *qDTY*_*3.1*_ + *qDTY*_*11.1*_	3987.25db	1911.91fe	2360.88ba	5269.26cb	600.30bd	—	—	—	—
H	*qDTY*_*1.1*_ + *qDTY*_*2.1*_ + BR	4577.64ed	1387.86c	3408.88ecd	5873.81ce	445.67b	3266.93bc	167.79cb	4336.75c	100.67a
I	*qDTY*_*1.1*_ + *qDTY*_*3.1*_ + BR	4443.41ced	1490.09c	4341.67e	7497.31 g	438.71bc	—	—	—	—
J	*qDTY*_*1.1*_ + *qDTY*_*11.1*_ + BR	3868.54cb	1953.22fd	3646.53ed	6572.26gfe	532.18bc	3091.47b	153.99cb	4434.72dc	184. 99b
K	*qDTY*_*2.1*_ + *qDTY*_*11.1*_ + BR	2616.89a	1389.51c	2369.08dba	4262.27ab	383.59b	—	—	—	—
L	*qDTY*_*1.1*_ + BR	—	—	—	—	—	—	95.94a	4318.98dc	71.53a
CH	Swarna	6171.13ed	641.62b	—	—	—	—	—	—	—
P1	Samba Mahsuri	3456.22ab	103.32a	1589.83a	3136.56a	0.0a	1124.17a	15.31a	4202.96dcb	0a
P2	IR55419-04	4383.55ced	2021.96fe	3168.96ecd	5889.33ce	814.73d	2803.41b	291.94d	3233.80a	1047.97d
F-value		6.81	35.44	5.67	18.76	4.49	12.02	8.19	6.06	64.76
p-value		0.0005	<0.0001	0.0013	<0.0001	<0.0001	<0.0001	<0.0001	0.0005	<0.0001

Means followed by the same letter (within a column) are not significantly different (F-test), NS: non-stress, RS: reproductive-stage drought stress, CH: drought-susceptible check, P1: recipient parent (no QTL), P2: donor parents (qDTY_1.1_ + qDTY_2_._1_ + qDTY_3_._1_ + qDTY_11_._1_ + BR), BR: blast resistance.

The mean grain yield of PLs with *qDTY*_*2.2*_ + *qDTY*_*4.1*_ (class C) is significantly higher than in lines with a single QTL in DS 2016 (Table 2). Among the lines with a single *qDTY*, lines with *qDTY*_*4.1*_ (class B) outperformed the lines with *qDTY*_*2.2*_ (class A) under RS and NS conditions (Table 2). In marker-assisted recurrent selection experiments, PLs with four *qDTYs* (*qDTY*_*1.1*_ + *qDTY*_*2.1*_ + *qDTY*_*3.1*_ + *qDTY*_*11.1*_) + blast resistance (class A) and two *qDTYs* (*qDTY*_*1.1*_ + *qDTY*_*11.1*_) + blast resistance (class J) yielded higher under RS and NS conditions than other PLs with two and three *qDTYs* (Table 3). Among the three *qDTY* PLs, lines having *qDTY*_*1.1*_ + *qDTY*_*2.1*_ + *qDTY*_*11.1*_ + blast resistance (class F) performed better under both conditions compared to other QTL combinations (Table 3). Among the Samba Mahsuri PLs with two *qDTYs*, *qDTY*_*1.1*_ + *qDTY*_*11.1*_ + blast resistance (class J) and *qDTY*_*1.1*_ + *qDTY*_*2.1*_ + blast resistance (class H) showed better yield advantage under both RS and NS conditions compared to other QTL class with two *qDTYs* (Table 3).

### Effect of QTL pyramiding on agronomic traits

The Samba Mahsuri PLs with either single or multiple *qDTYs* produced higher yield than the recipient parent in both marker-assisted backcross and marker-assisted recurrent selection experiments even under severe drought stress (Supplementary Fig. S5). The severity of drought stress can be assessed by the water table level^8,9^ (Supplementary Fig. S6). The performance of the most promising drought-tolerant PLs in the marker-assisted backcross experiments at IRRI (Philippines), Hyderabad (India) and in the marker-assisted recurrent experiment at IRRI (Philippines) is presented in Tables 4 and 5, respectively. These lines were selected after phenotypic selection, genotypic selection, selection based on grain type, and stable performance under RS and NS conditions. DTF of selected promising lines with *qDTY*_*2.2*_ + *qDTY*_*4.1*_ varied from 74 to 88 days and from 77 to 94 days under NS and RS conditions, respectively, at IRRI, Philippines (Table 4), and from 87 to 114 days and from 89 to 111 days under NS and RS conditions, respectively, at Hyderabad, India (Table 4). The PHT of selected promising lines with *qDTY*_*2.2*_ + *qDTY*_*4.1*_ did not vary much from that of Samba Mahsuri under RS and NS conditions (Table 4). The selected promising PLs in the marker-assisted recurrent selection experiment flowered earlier than the recipient parent but the variability of PLs ranged from 53 to 100 days in NS and from 72 to 119 days under RS (Table 5). The PHT of promising PLs in the marker-assisted recurrent selection experiment ranged from 86 to 122 cm and from 64 to 95 cm under NS and RS, respectively (Table 5). The performance of PLs is dependent on season and level of stress (Tables 4 and 5). The grain yield advantage of selected promising PLs over Samba Mahsuri in MAB and MARS experiments is shown in Supplementary Tables S1 and S2, respectively. The grain yield advantage ranged from 297 to 4232 kg ha^−1^ and from 127 to 1299 kg ha^−1^ under NS and RS, respectively, in MAB experiments. The grain yield advantage ranged from 1467 to 5312 kg ha^−1^ and from 81 to 2585 kg ha^−1^ under NS and RS, respectively, in MARS experiments except for the yield of IR 102818-10-266-3-2-2-6 and IR 102821-19-233-2-1-1-10 in DS 2014 and IR 102818-10-227-1-2-1-2 in WS 2014 under NS conditions.

**Table 4:** Performance of promising PLs with *qDTY*_*2.2*_ + *qDTY*_*4.1*_ in terms of data on days to 50% flowering (days), plant height (cm), and grain yield (kg ha^−1^) under reproductive-stage drought stress (RS) and irrigated non-stress (NS) conditions at IRRI, Philippines and Hyderabad, India.

Location	Season	Agronomic trait	Environment	IR 99734:1-33-69-1-39-6	IR 99734:1-33-69-1-12-8	IR 99734:1–33–69–1–12–9	IR 99734:1-33-304-1-5-8	IR 99734:1-33-304-1-5-10	Samba Mahsuri	Trial Mean	H
Philippines	DS 2016	DTF	NS	79	80	74	75	74	93	85 ± 4	0.73
			RS	80	82	77	77	79	—	82 ± 3	0.65
		PHT	NS	93	94	91	91	84	77	89 ± 1	0.74
			RS	71	71	71	71	56	—	66 ± 2	0.77
		GY	NS	5154	4682	4400	4400	4107	4951	4575 ± 184	0.70
			RS	296	272	219	340	250	0	222 ± 110	0.54
	WS 2015	DTF	NS	81	82	81	83	81	105	87 ± 1	0.83
			RS	84	86	79	88	81	130	98 ± 7	0.74
		PHT	NS	108	104	106	108	109	101	105 ± 4	0.85
			RS	85	74	85	80	71	74	71 ± 6	0.70
		GY	NS	4508	3847	4353	5404	4880	4044	4180 ± 569	0.75
			RS	498	278	204	175	190	48	114 ± 126	0.64
	DS 2015	DTF	NS	87	88	87	85	85	100	88 ± 1	0.88
		PHT	NS	92	84	92	94	92	85	86 ± 7	0.79
		GY	NS	9482	6176	6143	7586	7002	5325	6144 ± 1208	0.75
	WS 2014	DTF	NS	84	84	84	86	86	100	88 ± 2	0.84
		PHT	NS	103	103	103	103	103	95	100 ± 3	0.78
		GY	NS	3242	3073	3073	2842	2842	2137	3301 ± 488	0.72
	DS 2014	DTF	NS	87	88	88	83	83	102	84 ± 4	0.88
			RS	94	94	94	90	90	—	92 ± 4	0.73
		PHT	NS	93	91	91	87	87	87	91 ± 6	0.77
			RS	70	65	65	64	64	—	62 ± 5	0.68
		GY	NS	13985	10371	10371	10050	10050	9753	8133 ± 736	0.88
			RS	624	1109	1109	823	823	0	850 ± 556	0.78
	DS 2013	DTF	NS	78	78	78	81	81	89	83 ± 1	0.75
			RS	86	86	86	83	83	—	85 ± 2	0.67
		PHT	NS	79	79	79	82	82	80	81 ± 1	0.77
			RS	77	77	77	79	79	—	69 ± 3	0.64
		GY	NS	2549	2549	2549	1399	1399	2486	2376 ± 118	0.88
			RS	1299	1299	1299	1279	1279	0	1366 ± 485	0.73
	DS 2016	BLB score		7	7	7	1	1	7	5	
		Blast score		1	2	2	4	3	1	2	
Hyderabad	**Season**	**Agronomic trait**	**Environment**	**IR 99734:1-33-69-1-20-SM-1**	**IR 99734:1-33-69-1-9-SM-1**	**IR 99734:1-33-69-1-9-SM-5**	**IR 99734:1-33-304-1-5-SM-1**	**IR 99734:1-33-304-1-5-SM-5**	**Samba Mahsuri**	**Trial Mean**	
	WS 2015	DTF	NS	97	95	97	94	96	110	97 ± 2	0.90
			RS	97	111	96	95	107	—	97 ± 1	0.67
		PHT	NS	113	113	113	113	113	112	113 ± 4	0.50
			RS	60	58	58	54	56	—	56 ± 1	0.60
		GY	NS	7851	7586	8111	7130	6478	4166	7570 ± 581	0.86
			RS	610	723	536	419	559	0	658 ± 59	0.58
	DS 2015	DTF	NS	114	107	108	108	105	112	86 ± 3	0.95
		PHT	NS	86	83	88	81	85	79	89 ± 3	0.81
		GY	NS	5645	6012	6365	4788	6239	7403	5858 ± 880	0.51
	WS 2014	DTF	NS	90	90	90	87	87	101	88 ± 1	0.95
			RS	92	89	89	94	94	111	96 ± 1	0.82
		PHT	NS	106	96	96	96	96	86	97 ± 4	0.84
			RS	64	65	65	55	55	53	61 ± 2	0.77
		GY	NS	6407	6699	6699	6389	6389	4762	6285 ± 557	0.79
			RS	115	519	519	227	227	0	322 ± 35	0.57

NS: Non stress, RS: Reproductive stage drought stress, DTF: Days to 50% flowering, PHT: Plant height, GY: Grain yield, H: Heritability, BLB and Blast score: 0: highly resistant, 1–2: resistant, 3–4: moderately resistant, 5–6: moderately susceptible, 7–8: susceptible, 9: highly susceptible

**Table 5:** Performance of promising PLs with four *qDTYs* (*qDTY*_*1.1*_, *qDTY*_*2.1*_, *qDTY*_*3.1*_, and *qDTY*_*11.1*_) in terms of data on days to 50% flowering (days), plant height (cm), and grain yield (kg ha^−1^) under reproductive-stage drought stress (RS) and irrigated non-stress (NS) conditions.

Season	Agronomic trait	Environment	IR 102818–10–227–1–2–1–2	IR 102818-10-227-1-2-1-6	IR 102818-10-227-1-2-1-9	IR 102821-19-233-2-1-1-10	IR 102818-10-276-1-2-2-9	IR 102818-10-276-1-2-2-11	IR 102818-10-266-3-2-2-6	Samba Mashuri	Trial Mean	H
			*DTY*_*1.1*_ + *DTY*_*11.1*_	*DTY*_*1.1*_ + *DTY*_*11.1*_	*DTY*_*1.1*_ + *DTY*_*11.1*_	*DTY*_*1.1*_ + *DTY*_*2.1*_ + *DTY*_*3.1*_ + *DTY*_*11.1*_	*DTY*_*1.1*_ + *DTY*_*2.1*_ + *DTY*_*11.1*_	*DTY*_*1.1*_ + *DTY*_*2.1*_ + *DTY*_*11.1*_	*DTY*_*1.1*_ + *DTY*_*2.1*_			
DS 2016	DTF	NS	56	54	57	53	62	61	62	76	80 ± 2	0.88
		RS	72	78	75	74	84	85	90	—	88 ± 4	0.79
	PHT	NS	102	101	99	86	103	99	100	81	100 ± 3	0.85
		RS	76	81	69	81	73	76	64	—	73 ± 6	0.73
	GY	NS	4053	3759	4485	3561	4779	4440	4933	4203	4370 ± 388	0.80
		RS	304	343	278	541	168	183	115	0	150 ± 73	0.65
WS 2015	DTF	NS	99	97	100	85	92	97	97	108	97 ± 2	0.78
		RS	117	119	116	81	118	117	118	131	116 ± 4	0.85
	PHT	NS	122	111	103	103	114	120	103	87	116 ± 8	0.88
		RS	95	94	87	85	91	80	81	74	85 ± 6	0.78
	GY	NS	3541	2867	2592	4548	4191	4820	3954	1124	3186 ± 687	0.80
		RS	377	252	405	285	96	110	262	15	126 ± 101	0.60
DS 2015	DTF	NS	84	80	83	87	87	88	89	99	91 ± 15	0.86
	PHT	NS	103	107	104	86	95	102	106	68	99 ± 8	0.88
	GY	NS	5827	6998	8449	5634	6731	6307	6617	3137	5824 ± 1315	0.79
WS 2014	DTF	NS	91	91	91	84	94	94	94	103	93 ± 3	0.82
	PHT	NS	121	121	121	104	122	122	112	95	109 ± 5	0.85
	GY	NS	1313	1313	1313	2332	4246	4246	3157	1590	3131 ± 1126	0.80
DS 2014	DTF	NS	74	74	74	78	79	79	89	102	88 ± 6	0.88
		RS	77	77	77	80	80	80	94	112	92 ± 3	0.74
	PHT	NS	107	107	107	101	115	115	92	84	105 ± 6	0.88
		RS	81	81	81	80	79	79	76	54	79 ± 6	0.66
	GY	NS	6283	6283	6283	3466	7894	7894	3231	3456	4389 ± 1186	0.72
		RS	1825	1825	1825	1857	2688	2688	1618	103	1751 ± 375	0.55
BLB Score			7	7	7	7	7	7	7	7	6	
Blast Score			1	0	0	1	0	2	0	1	1	

NS: Non stress, RS: Reproductive stage drought stress, DTF: Days to 50% flowering, PHT: Plant height, GY: Grain yield, H: Heritability, BLB and Blast score: 0: highly resistant, 1–2: resistant, 3–4: moderately resistant, 5–6: moderately susceptible, 7–8: susceptible, 9: highly susceptible.

### Background genotyping and genetic similarity studies of MAB lines

**Figure 1:**
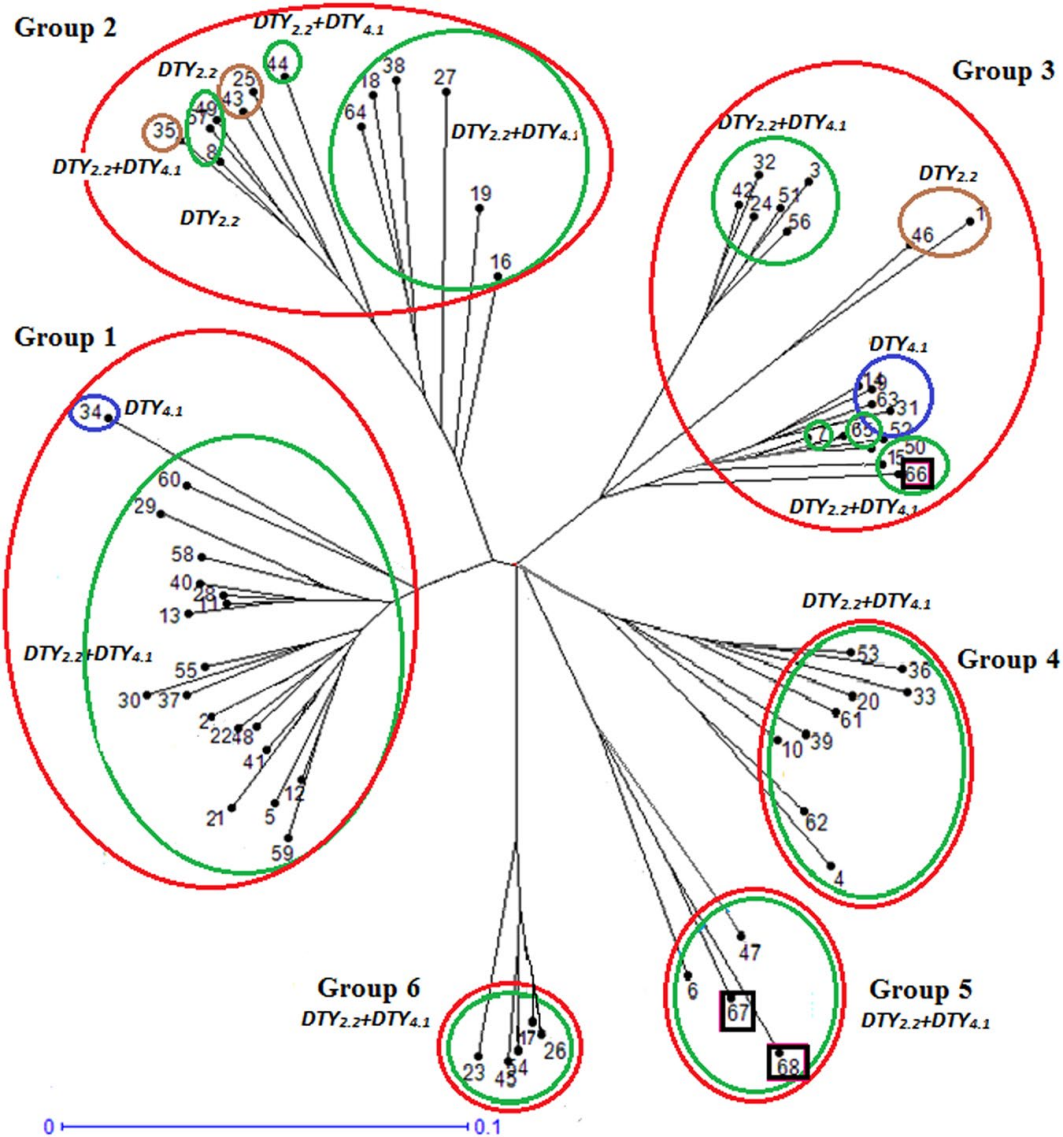
Unrooted neighbor-joining dendrogram of 65 PLs, donor (IR 87728-75-B-B), recipient (Samba Mahsuri), and drought-tolerant check (IR 87707-445-B-B) using SSR fingerprint data sets. Green color circle: PLs with *qDTY *_*2.2*_+ *qDTY*_*4.1*_, blue color circle: PLs with *qDTY*_*4.1*_, brown color circle: PLs with *qDTY*_*2.2*_, red color circle: group, black color circle: donor, recipient parent, and drought-tolerant check, *data from one PL only in DS 2016. The numeric number represents the PLs. The designation details of the PLs are shown in Supplementary Table S3.

**Figure 2:**
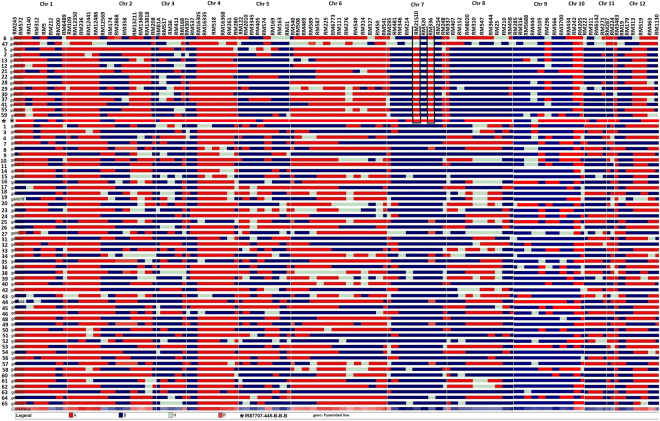
Graphical representation of allelic distribution of 65 PLs (numerical number on left hand side) and drought-tolerant check (*IR 87707-445-B-B) across 12 chromosomes using Graphical Genotypes software (GGT 2.0). A: donor allele, B: recipient allele, H: heterozygous allele, R: recombinant allele. The designation details of the PLs (numerical number on left hand side) are shown in Supplementary Table S3.

The PLs with *qDTYs qDTY*_*2*__.__*2*_ + *qDTY*_*4*__.__*1*_, *qDTY*_*2*__.__*2*_, and *qDTY*_*4*__.__*1*_, showed a consistent grain yield advantage under drought stress and non-stress conditions over Sambha Mahsuri. However, variability in yield among the pyramided lines with the same QTL combination was observed (Supplementary Table S3). To study the allelic constitution of PLs with the same QTLs combination but showing variation in yield, background genotyping was carried out on the 65 selected PLs, two parents, and one check (IR 87707-445-B-B-B) in WS 2015 using 99 polymorphic markers well distributed across the genome. Allele statistics for donor, recipient, heterozygous, and recombinant allele frequency were calculated (Supplementary Table S3). Hierarchical clustering based on the Unweighted Pair Group Method with Arithmetic Mean (UPGMA) using DARwin 6.0.013 (Dissimilarity Analysis and Representation win^35^) divided the PLs into six groups (Fig. 1). These six groups were created with 19, 13, 18, 9, 4, and 5 PLs in groups 1, 2, 3, 4, 5, and 6, respectively. PL 6 (IR 99734:1-33-304-1-5-8) and PL 47 (IR 99734:1-33-304-1-5-10) grouped with donors IR 87728-75-B-B and IR 87707-445-B-B-B (IR64 introgressed line with *qDTY*_*2.2*_ and *DTY*_*4*__.__*1*_, released in India, Nepal, and Myanmar). Samba Mahsuri was classified in Group 3. PLs with *qDTYs* in one group were grouped together and the mean GY of each group class was calculated under RS and NS conditions (Supplementary Table S4). The PLs in Groups 1 and 5 showed a higher grain yield advantage under RS in WS 2015 and DS 2016 (Supplementary Table S4). Visualization analysis of molecular marker scores was done using GGT 2.0 software^36^ and colored chromosome bar segments representing allelic distribution are shown in Fig. 2. The PLs in Groups 1 and 5 that had shown better performance across seasons were reported to have a conserved region near RM21510 on chromosome 7. This region is also present in the IR64 introgressed line with *qDTY*_*2.2*_ and *qDTY*_*4.1*_, IR 87707-445-B-B-B (Fig. 2). The PLs with a conserved genetic region on chromosome 7 had shown a yield advantage over PLs without the conserved genetic region under NS and severe reproductive-stage drought stress (Supplementary Table S5). Supplementary Fig. S6 validates the severity of reproductive-stage drought stress in WS 2015 and DS 2016.

### Epistasis interaction

**Figure 3:**
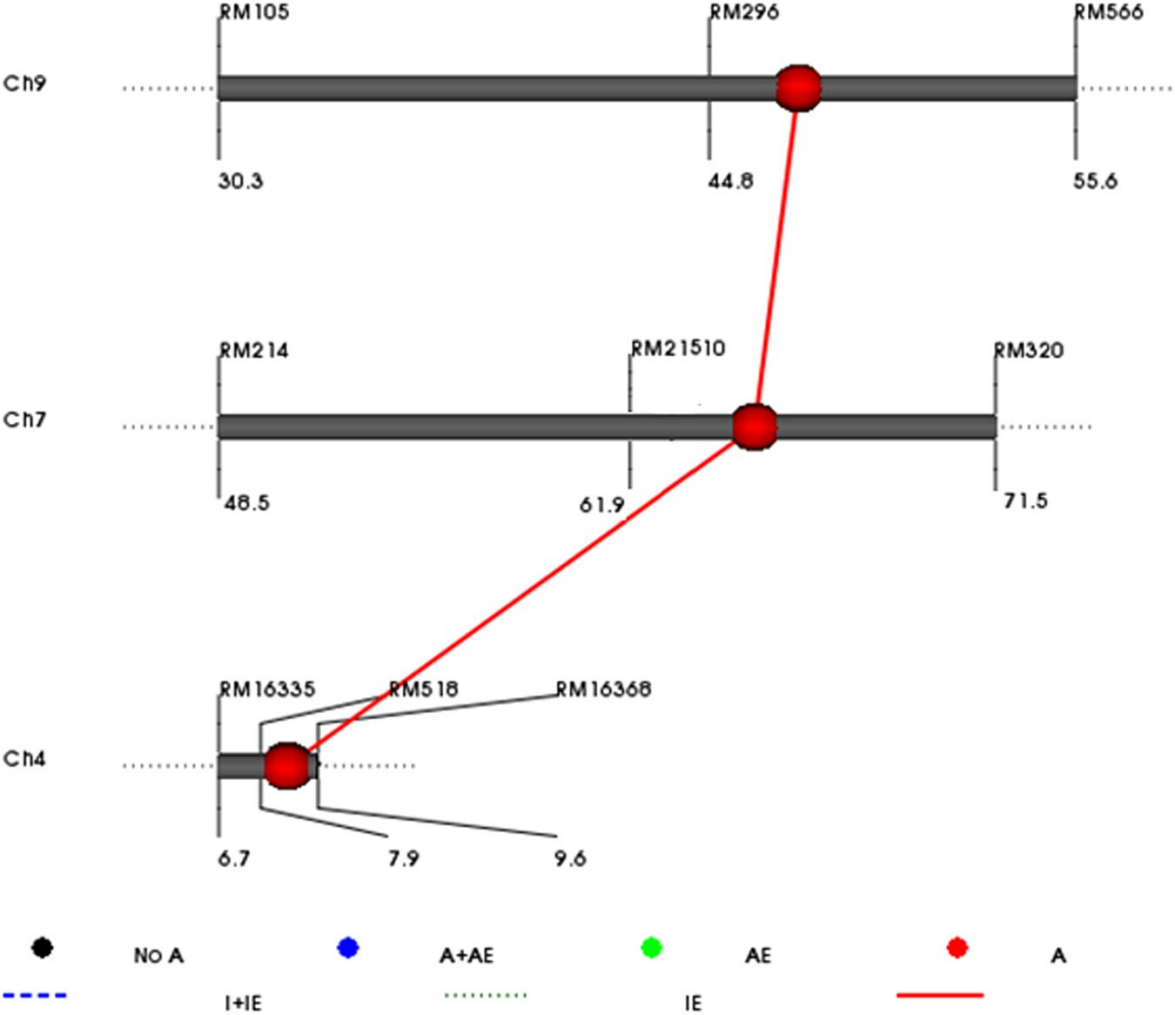
Epistasis interactions among *qDTY*_*4.1*,_ *qDTY*_*7.1*,_ and *qDTY*_*9.1*_ in marker-assisted backcrossing experiment. A: additive effect, AE: additive × environment interaction effect, I: epistatic main effect, IE: epistasis × environment interaction effect, with only epistatic main effect (I).

PLs with the same QTL combinations showing variability for grain yield, grouping of all selected high-yielding promising PLs in one group (Fig. 1) and the GGT map showing a conserved region in all the selected PLs on chromosome 7 (Fig. 2), supports the possibility of interaction among contributing alleles/QTLs. Two dimensional genome scan revealed epistasis interaction of *qDTY*_*7.1*_ (RM21510-RM320, on chromosome 7) with two loci, *qDTY*_*4.1*_ (RM518-RM16368, on chromosome 4) and *qDTY*_*9.1*_ (RM296-RM566, on chromosome 9) with an additive by additive effect of 18.7 (p ≤ 0.01) and 33.5% (p ≤ 0.0001) of the population mean, respectively (Supplementary Table S6, Fig. 3). The heritability of the additive x additive effect ranged from 1.4 (*qDTY*_*7.1*_ with *qDTY*_*4.1*_) to 6.4 (*qDTY*_*7.1*_ with *qDTY*_*9.1*_). The PLs with the donor (IR 87728-75-B-B) allele at *qDTY*_*4.1*_, *qDTY*_*7.1*_ and *qDTY*_*9.1*_ loci showed the higher mean grain yield under stress over the lines not possessing the donor allele for three loci. It is worth mentioning that the *qDTY*_*7.1*_ and *qDTY*_*9.1*_ on chromosome 9 were not targeted in the introgression program but detected during epistasis interaction study.

## Discussion

In the present study, bacterial blight-/blast-tolerant, lowland-adapted but drought-susceptible, high grain and cooking quality line, Samba Mahsuri was used as a recipient parent to improve its yield under drought. Pre-breeding lines possessing major-effect *qDTYs* showing high grain yield under drought were used as donor parents. The yield advantage under drought of PLs possessing earlier identified large and consistent-effect *qDTYs*, *qDTY*_*2.2*_ and *qDTY*_*4.1*_ (IR64 background^13^), *qDTY*_*1.1*_ (Swarna, IR64, and MTU1010 background^6^), *qDTY*_*2.1*_ (Swarna background^5^), and *qDTY*_*3.1*_ (Swarna and TDK1 background^5,17^) in current marker-assisted selection and recurrent selection breeding programs indicates the suitability of these loci in improving drought tolerance in the Samba Mahsuri background.

The development of PLs with positive interaction of QTLs has provided yield advantage of 1.0–1.2 t ha^−1^ under RS as well as stable grain yield under NS. The increase in yield under RS of PLs possessing single or combinations of *qDTYs* indicate the suitability of the *qDTYs* in increasing yield under RS. The increase in yield of single and multiple *qDTY* PLs in different backgrounds reported earlier (Vandana, IR64, Swarna, TDK1, MTU1010^37^, and MRQ74^23^) as well as the present study validates the success of QTL introgression in increasing yield under drought stress. These successful examples should encourage use of *qDTYs* in breeding programs targeting yield improvement under drought.

As shown earlier in IR64 background (Swamy *et al*.^13^), in the current study also, *qDTY*_*2.2*_*, qDTY*_*4.1*_ combination showed higher yield advantage under drought over single QTLs in Samba Mahsuri background indicating the effectiveness as well as positive interactions between these two QTLs in multiple genetic backgrounds. The release of IR64 PLs with *qDTY*_*2.2*_ + *qDTY*_*4.1*_ in India, Nepal, and Myanmar, validate the effect of these QTLs in reducing yield loss under reproductive-stage drought stress in variable environments (Sandhu *et al*.^38^).

This is the first study in rice comparing the yield advantage under severe reproductive stage drought achieved through MAS and MARS strategies. Severe and cyclic drought stress exposure to the population in the present study assisted in identifying true drought-tolerant lines^39^ with different growth duration^40^. The grain yield advantage in selected promising PLs over the recipient parent is high in MARS compared to MAS. The superiority of MARS lines may be because of accumulation of higher proportion of drought tolerant alleles during recurrent selection process as compared to the lines developed through MAS. MARS in sweet corn^41,42^, soybean and sunflower^43^, maize^18,21,43–47^, wheat^48^, and cucumber^24,25,49^ has proven to be effective in increasing the frequency of favorable alleles with improvement in grain yield, grain yield-related traits, and drought tolerance.

Drought stress in general occurs together with a high prevalence of biotic stresses such as bacterial blight, blast, and brown spot. The development of high-yielding drought-tolerant rice varieties with tolerance of biotic stress could considerably help to control heavy yield losses. However, very few molecular breeding studies have been undertaken to study the combined effect of abiotic and biotic stress tolerance simultaneously in mapping populations. The present study reports an integrated strategy of QTL pyramiding to develop PLs with high grain yield under reproductive-stage drought stress together with tolerance of biotic stress. We observed that early generation-systematic screening of large F_2_ population for biotic stress tolerance may provide opportunity to select lines for the second targeted trait (high grain yield under RS) in reduced time span. The developed PLs having tolerance to both biotic and abiotic stresses may help to identify, exploit and understand the mechanism of potential QTLs/genes providing tolerance to multiple stresses. It may also serve as useful resource for crop improvement program directed toward improving agronomic traits and multiple stress resistance.

The GY advantage of the PLs with *qDTY*_*1.1*_ and *qDTY*_*11.1*_ either single or in combination with other QTLs under RS and NS indicates the superiority of *qDTY*_*1.1*_ and *qDTY*_*11.1*_ in marker-assisted introgression programs over other QTLs. *qDTY*_*1.1*_ has been reported to be associated with increased yield under multiple conditions (dry direct seeded, drought, non-stress) and multiple backgrounds (Swarna, IR64, MTU1010)^6,12,50^. Dixit *et al*.^17^ reported the performance of *qDTY*_*11.1*_ in a Swarna background. In this study, across the PLs with two, three, four QTLs combinations, *qDTY*_*1.1*_ showed positive interaction with *qDTY*_*11.1*_ as lines possessing these two combinations of QTLs outyielded lines with other QTLs combinations. The study indicates that the breeding programs targeting MAS of *qDTY*_*1.1*_ should also include *qDTY*_*11.1*_ for higher yield advantage under both NS and RS.

The use of donors having *qDTY*_*9.1*_ (*qDTY*_*9.1*_; IR 77298-5-6-B-18)^13^ supported the presence of *qDTY*_*9.1*_ in the Samba Mahsuri background in the current MAS QTL introgression program. The presence of *qDTY*_*9.1*_ in Samba Mahsuri (Fig. 3) and IR64 background^13^ under reproductive-stage drought stress conditions indicates the effectiveness of the genetic region in different backgrounds. The stable performance of selected PLs in the Philippines and Hyderabad, India (Supplementary Table S1), indicates the effectiveness of *qDTY*_*9.1*_ in different backgrounds (Samba Mashuri, IR64) and environments.

The presence of the conserved allelic region on chromosome 7 (marker RM21510) in selected promising PLs showed higher yield advantage and its absence showed lower yield advantage under RS even if the other *qDTYs* were present (Fig. 2, Supplementary Table S3). The introgressed QTLs do not explain the entire phenotypic variation of the PLs for GY, this potentially indicates the importance of capturing the positive digenic interaction of *qDTY*_*7.1*_ with *qDTY*_*4.1*_ and *qDTY*_*9.1*_ (Fig. 3, Supplementary Table S6). The epistasis interactions of these loci suggest their importance in elucidating the genetic basis of GY in high yielding PLs under RS. Understanding the genetic composition of these identified loci and their effect on GY may allow us to use these loci to achieve maximum GY advantage under RS. Earlier, Dixit *et al*.^22^ reported the positive interaction of *qDTY*_*2.3*_ and *qDTY*_*3.1*_ with *qDTY*_*12.1*_ and a significant effect on grain yield increase under RS drought stress. Unlike Dixit *et al*.^22^ where in interactions between two identified QTLs were reported, in the present study, even if no QTLs for grain yield under drought near RM10 on chromosome 7 has been detected, the effect of the interaction of this region with *qDTY*_*4.1*_ and *qDTY*_*9.1*_ has significantly enhanced the yield under drought. In an earlier study, genetic loci for grain yield under drought stress, plasticity for root dry weight and total water uptake, and total root length were identified^49^ located near *qDTY*_*7.1*_ and *qDTY*_*9.1*_, respectively. The broad understanding of the interactions identified in the present study and introgression of such specific combinations of a few QTLs may be more effective in increasing yield than random combinations of many QTLs. The complexity of effect and interaction among the most favorable alleles or haplotypes controlling the trait of interest, differential expression of expressed regions under variable conditions, and increase in frequency of positive alleles over negative contributing alleles may be responsible for the differential behavior of PLs having the same QTLs.”

Previously identified QTLs and genes in *qDTY*_*4.1*,_ *qDTY*_*7.1*_ and *qDTY*_*9.1*_ may provide insights as to why the QTLs identified in the present study contribute to grain yield advantage under RS. The QTLs associated with germination^51^, panicle length (sp2(t))^52^, tiller number (tp7(b))^53^, days to flowering (dth7.1)^54^ and rice stripe virus resistance (Rurm1)^55^ were identified in *qDTY*_*7.1*_ region. Various genes related to stress-signaling, stress-responses and signaling, growth and development processes, and hormonal regulation and transcription factors were reported within *qDTY*_*4.1*_ and *qDTY*_*9.1*_ by Swamy *et al*.^13^. The upstream region of *qDTY*_*7.1*_ (15603452-18640879 bp) found to be associated with the gene responsive to phosphate uptake efficiency (Li *et al*. 2014^56^) and downstream region with gene conferring resistance to blast and bacterial late blight (*Calmodulin-Binding Transcription Factor; oscbt*; 18865438-18878266 bp; http://qtaro.abr.affrc.go.jp/ogro/entry/show/660; Koo *et al*. 2008^57^). There could be a probability that the genomic region (15414191-18878266) on chromosome 7 may be involved in providing tolerance to multiple stresses involving increasing nutrient uptake to increase yield under drought and biotic stress resistance. Further detailed studies on targeted genomic region on chromosome 7 may reveal additional information.

## Conclusions

The results reported in the present study indicate the effectiveness of introgressed QTLs, interaction of QTLs with other loci, to further enhance GY under reproductive-stage drought stress while following MAS and MARS approaches. The GY advantage achieved is more in MARS than in MAS. Drought-tolerant Samba Mahsuri PLs with a grain yield advantage of 0.5–1.0 t ha^−1^ under reproductive-stage drought stress were developed. The PLs showing similar grain type and plant type as Samba Mahsuri together with the tolerance to biotic and abiotic stress may act as a candidate to replace the variety Samba Mahsuri. The positive interaction of introgressed QTLs with other QTL/genetic region and genetic background as reported in the present study could be one of the possible reason for the variable effect of introgressed QTLs in PLs. Identification of these positive interactions, allele mining and complete sequencing of promising PLs could reduce to a certain extent QTL x genetic background interactions often observed under RS.

## Materials and Methods

### Plant material, phenotyping, and management

The study was conducted at the International Rice Research Institute (IRRI), Los Baños, Laguna, Philippines. To evaluate the effect of QTLs that had earlier shown an effect in the IR64 background under drought, two approaches were used. The popular rice variety Samba Mahsuri (occupying 3.3% of rice growing area in India), was used as a recipient to develop mapping populations through marker-assisted breeding and marker-assisted recurrent selection approaches. Samba Mahsuri is a medium-tall (90–100 cm) variety with 140–145 days duration, superfine grain with excellent grain and cooking quality, hulling percentage of 70%, head rice recovery of 75%, kernel length of 5.45 mm, kernel breadth of 1.97 mm, L/B ratio of 2.7, elongation ratio of 1.85, alkali spreading value of 5.5, and amylose content of 24.8%. Samba Mahsuri is a very popular variety, preferred for its fine slender premium grain and excellent cooking quality. The drought-tolerant *indica* rice varieties/pre-breeding lines developed at IRRI, IR 87728-75-B-B possessing *qDTY*_*2.2*_ and *qDTY*_*4.1*_ and IR55419-04 possessing *qDTY*_*1.1*_*, qDTY*_*2.1*_*, qDTY*_*3.1*_, and *qDTY*_*11.1*_, were used as donors in marker-assisted backcross and marker-assisted recurrent selection approaches, respectively. The scheme of developing the Samba Mahsuri PLs and number of selected plants in each subsequent generation using MAB and partial MARS approaches is shown in Supplementary Figs S2 and S3, respectively. The lines were screened under lowland transplanted control and lowland reproductive-stage drought stress conditions. Screening of PLs was conducted using an α-lattice or randomized complete block design (RCBD) or augmented RCBD along with drought-tolerant and susceptible checks and donor/recipient parents in 1–4-row plots 3–5 m in length, with 0.20–0.25-m row-to-row spacing and 2.0–2.5 g seed per linear meter. For all the trials, the seeds were sown in a raised bed nursery and 21–25-day-old seedlings were transplanted to the main field. Inorganic fertilizers NPK (nitrogen, phosphorus, and potassium) were applied @ 120:30:30 kg ha^−1^. Weeds, insect pests, and snails management was done as described by Venuprasad *et al*.^5^. For non-stress, the trials were conducted under irrigated, transplanted, flooded, puddled, and anaerobic conditions with no drought stress. The reproductive-stage drought stress experiments were carried out in an automated rainout shelter facility at IRRI.

For reproductive-stage stress, transplanted experiments were maintained as described by Sandhu *et al*.^58^. The drought stress was initiated at 32 days after transplanting. After the inception of the stress, the soil water potential was measured using tensiometers (30 cm depth) in DS 2013 and DS 2014 (only). The plots in the reproductive stage drought stress treatments were rewatered when the soil water potential dropped to −50 to −70 kPa (tensiometer). The decline in water table depth was measured on a daily basis with a meter scale inserted into a 1.1-m polyvinyl chloride pipe in the experimental fields at regular intervals in all RS treatments. The pipes were placed at 1.0-m depth with 10 cm of pipe remaining above the soil surface. The plots were rewatered when water table level reached 100 cm below the soil surface and most lines were wilted and exhibited severe leaf drying. This cyclic reproductive stage drought stress allows the effective screening of broad range growth duration PLs^40^.

Data on days to 50% flowering (days) were recorded when 50% of the plants in the plot started flowering. At maturity, when 80–85% of the panicles turned golden yellow, plant height (cm) was measured as the mean height of three plants per plot from the base to the tip of the tallest panicle. The grains were harvested from each plot, dried to a moisture content of 14%, and weighed. Grain yield data were normalized as per moisture content for yield computation (kg ha^−1^). Visual observation on grain type similar to that of the recipient parent was made in the field per plot during plot selection and per plant during panicle selection. Blast (caused by *Magnaporthe oryzae)* and bacterial blight (caused by *Xanthomonas oryzae* pv*. Oryzae*) screening was done twice first at F_2_ and then at F_8_ stage. Mixed inoculum for races present in Philippines was used for blast inoculation. Genes *pik-s, pi2, pi5(t), pi9* showed resistance against the mixed inoculum. For bacterial blight inoculation race 1 (*PXO61*) and race 2 (*PXO86*) was used. Inoculation and scoring for bacterial blight were done at maximum tillering stage as described by Kauffman *et al*.^59^. Inoculation for blast screening was done 10 days after seeding in the blast nursery and, after an exposure of 22 days, scoring was done based on the SES scale (0: highly resistant, 1–2: resistant, 3–4: moderately resistant, 5–6: moderately susceptible, 7–8: susceptible, 9: highly susceptible)^60,61^. Phenotypic selection was used for biotic screening. Selection for resistance genes against blast and bacterial blight was not targeted in the present study.

### Genotyping

Molecular marker work was carried out in the Molecular Marker Application Laboratory of IRRI. Fresh young leaves from all lines were collected, freeze-dried, and the DNA extracted using the modified CTAB protocol^62^. A total of 112 SSR markers linked to two *qDTY* regions were tested for polymorphism in the marker-assisted breeding experiment. The markers linked to *qDTY*_*2.2*_ (RM236, RM279, RM109) and *qDTY*_*4.1*_ (RM335, RM551, RM518) were found to be polymorphic in the Samba Mahsuri background and were used for foreground selection. The lines having the same QTL combination were found to be segregating for yield components. To study the allelic pattern of these lines, a total of 650 SSR markers distributed across the rice genome were tested for polymorphism. A total of 99 polymorphic markers were used for the background study. In the marker-assisted recurrent selection experiment, a total of 200 markers linked to four *qDTY* regions were tested for polymorphism. Foreground and recombinant selection were carried out using RM212 and RM486 (chromosome 1), RM525 and RM221 (chromosome 2), RM16 and RM520 (chromosome 3), and RM287 (chromosome 11) polymorphic markers. Amplification was carried out using polymerase chain reaction (PCR) and PCR products were resolved in non-denaturing polyacrylamide gel electrophoresis (6% or 8%) depending on product size. Gels were stained with SYBR SafeTM DNA, viewed after 20 min, and DNA profiles were scored based on parent allelic profile. Stepwise selection involving phenotyping and genotyping was used to select and advance the desirable plants in every generation.

### Statistical analysis

Mean and standard error of difference of the experiments with alpha lattice were calculated using the linear mixed model of CROPSTAT version 7.2.3 considering replications and blocks within replication as random effects and lines as fixed effects.

$${y}_{ijk}=\mu +{g}_{i}+{r}_{j}+{b}_{lj}+{e}_{ijk},$$

where *μ* is the overall mean, *g*_*i*_ is the effect of the *i*^*th*^ genotype, *r*_*j*_ is the effect of the *j*^*th*^ replicate, *b*_*lj*_ is the effect of the *l*^*th*^ block within the *j*^*th*^ replicate, and *e*_*ijk*_ is the error.

For the RCBD, the model used was:

$${y}_{ijk}=\mu +{g}_{i}+{r}_{j}+{e}_{ijk}$$

where *μ* is the overall mean, *g*_*i*_ is the effect of the *i*^*th*^ genotype, *r*_*j*_ is the effect of the *j*^*th*^ replicate, and *e*_*ijk*_ is the error.

For the augmented RCBD, the model used was:

$${y}_{ijk}=\mu +{g}_{i}+{b}_{l}+{e}_{ilk}$$

where *μ* is the overall mean, *g*_*i*_ is the effect of the i^th^ genotype, *b*_*l*_ is the effect of the *l*^*th*^ block, and *e*_*ilk*_ is the error.

### Class analysis for *qDTY* PLs

Class analysis for *qDTY* PLs was performed using SAS v9.2 (SAS Institute Inc. 2009), considering the effects of QTLs and genotypes within the QTL as fixed effects and replicate and blocks within replicate as random effects. The model used to see the performance *y*_*ijkl*_ of the *j*^*th*^ genotype nested within the *i*^*th*^ QTL class in the *l*^*th*^ block within the k^th^ replicate is as follows:

$${y}_{ijkl}=\mu +{r}_{k}+b{(r)}_{kl}+{q}_{i}+g{(q)}_{ij}+{e}_{ijkl}$$

where *μ* is the population mean, *r*_*k*_ is the effect of the *k*^*th*^ replicate, *b(r)*_*kl*_ + *q*_*i*_ is the effect of the *l*^*th*^ block within the *k*^*th*^ replicate, *q*_*i*_ is the effect of the *i*^*th*^ QTL, *g(q)*_*ij*_ is the effect of the *j*^*th*^ genotype nested within the *i*^*th*^ QTL, and *e*_*ijkl*_ is the error^63^. ANOVA and F test using SAS v9.2 (SAS Institute Inc. 2009) were used to see whether the QTL classes differed significantly from each other.

### Graphical representation of the genome

Graphical representation of molecular marker data was performed using the software Graphical Genotypes (GGT 2.0)^36^. The homozygous donor allele, homozygous recipient allele, heterozygous allele, and recombinant allele were scored as ‘A’, ‘B’, ‘H’, and ‘R’, respectively. The estimated proportion of the A, B, H, and R alleles in each PL was calculated using GGT 2.0.

### Diversity studies of PLs segregating for grain yield

DARwin 6.0.013 software was used to compute a pairwise distance matrix by calculating a dissimilarity matrix^35^. An Unweighted Pair Group Method with Arithmetic Mean (UPGMA) followed by bootstrap analysis with 1000 permutations was used to construct a neighbor joining tree.

### Q x Q interactions

Detection of QTL x QTL interactions in the marker-assisted pyramided population was performed in QTL Network 2.1 based on a mapping methodology summarized by Yang *et al*.^64^. A two-dimensional genome scan with 1000 permutation was performed to identify additive x additive interaction. Determination of the QTLs intervals, detection of Q x Q interactions, and their additive x additive effect were considered as significant at P ≤ 0.01.

## Electronic supplementary material

Supplementary Information 
