# Supplementary material for: Positive interactions of major-effect QTLs with genetic background that enhances rice yield under drought
Source: Sci Rep. 2018 Jan 26;8:1626. doi: 10.1038/s41598-018-20116-7 (PMC5786057; doi:10.1038/s41598-018-20116-7)
Supplement: Supplementary file 1 — Supplementary Information [file 41598_2018_20116_MOESM1_ESM.doc]

**Title Page:**

**Positive interactions of major-effect QTLs with genetic background that enhances rice yield under drought**

## Nitika Sandhu^1^, Shalabh Dixit^1^, B.P. Mallikarjuna Swamy^1^, Prashant Vikram^2^, Challa Venkateshwarlu^1^*,* Margaret Catolos^1^, Arvind Kumar^1^*

*^1^International Rice Research Institute, DAPO BOX 7777, Metro Manila, Philippines*

*^2^ International Maize and Wheat Improvement Center (CIMMYT), Mexico*

*Correspondence to [a.kumar@irri.org](mailto:a.kumar@irri.org)

**Supplementary Table S1.** Grain yield advantage (Kg ha^-1^) of promising PLs with *DTY_2.2_* + *DTY_4.1_* over Samba Mahsuri under reproductive stress and irrigated non stress conditions at Philippines and Hyderabad.

| **Location** | **Designation** | **DS 2016** | **WS 2015** | | **DS 2015** | **WS 2014** | | **DS 2014** | | **DS 2013** |
| --- | --- | --- | --- | --- | --- | --- | --- | --- | --- | --- |
|  |  | RS | NS | RS | NS | NS | RS | NS | RS | RS |
| **Philippines** | IR 99734:1-33-69-1-39-6 | 296 | 464 | 450 | 4158 | 1105 | - | 4232 | 624 | 1299 |
|  | IR 99734:1-33-69-1-12-8 | 272 | - | 230 | 851 | 936 | - | 618 | 1109 | 1299 |
|  | IR 99734:1-33-304-1-5-8 | 340 | 1360 | 127 | 2261 | 705 | - | 297 | 823 | 1279 |
|  | IR 99734:1-33-69-1-12-9 | 219 | 309 | 156 | 819 | 936 | - | 618 | 1109 | 1299 |
|  | IR 99734:1-33-304-1-5-10 | 250 | 836 | 142 | 1678 | 705 | - | 297 | 823 | 1279 |
|  | Samba Mahsuri* | 0 | 4044 | 48 | 5325 | 2137 | - | 9753 | 0 | 0 |
|  | Trial Mean | 222±110 | 4180±569 | 114±126 | 6144±1208 | 3301±488 | - | 8133±736 | 850±556 | 1366±485 |
| **Hyderabad** | IR 99734:1-33-69-1-20-SM-1 | - | 3685 | 610 | - | 1884 | 111 | - | - | - |
|  | IR 99734:1-33-69-1-9-SM-1 | - | 3420 | 723 | - | 2176 | 515 | - | - | - |
|  | IR 99734:1-33-69-1-9-SM-5 | - | 3945 | 536 | - | 2176 | 515 | - | - | - |
|  | IR 99734:1-33-69-1-25-SM-5 | - | 3187 | 901 | - | 1977 | 366 | - | - | - |
|  | IR 99734:1-33-304-1-5-SM-1 | - | 2964 | 419 | - | 1866 | 223 | - | - | - |
|  | IR 99734:1-33-304-1-5-SM-5 | - | 2312 | 559 | - | 1866 | 223 | - | - | - |
|  | Samba Mahsuri* | - | 4166 | 0 | - | 4762 | 0 | - | - | - |
|  | Trial Mean | - | 7570±581 | 658± | - | 6285±557 | 332±35 | - | - | - |

*GY (Kg ha^-1^) for Samba Mahsuri, For PLs: GY advantage (Kg ha^-1^)

**Supplementary Table S2.** Grain yield advantage (Kg ha^-1^) of promising PLs with *DTY_1.1_*, *DTY_2.1_*, *DTY_3.1_,* and *DTY_11.1_* over Samba Mahsuri under reproductive stress and irrigated non stress conditions at IRRI, Philippines.

|  |  | **DS 2016** | **WS 2015** | | **DS 2015** | **WS 2014** | **DS 2014** | |
| --- | --- | --- | --- | --- | --- | --- | --- | --- |
| Designation | QTLs | RS | NS | RS | NS | NS | NS | RS |
| IR 102818-10-227-1-2-1-2 | *DTY_1.1_* + *DTY_11.1_* | 304 | 2417 | 362 | 2690 | 277 | 2827 | 1722 |
| IR 102818-10-227-1-2-1-6 | *DTY_1.1_* + *DTY_11.1_* | 343 | 1742 | 237 | 3861 | - | 2827 | 1722 |
| IR 102818-10-227-1-2-1-9 | *DTY_1.1_* + *DTY_11.1_* | 278 | 1467 | 390 | 5312 | - | 2827 | 1722 |
| IR 102821-19-233-2-1-1-10 | *DTY_1.1_* + *DTY_2.1_* + *DTY_3.1_* + *DTY_11.1_* | 541 | 3424 | 270 | 2497 | 742 | 10 | 1754 |
| IR 102818-10-276-1-2-2-9 | *DTY_1.1_* + *DTY_2.1_* + *DTY_11.1_* | 168 | 3067 | 81 | 3594 | 2656 | 4438 | 2585 |
| IR 102818-10-276-1-2-2-11 | *DTY_1.1_* + *DTY_2.1_* + *DTY_11.1_* | 183 | 3696 | 95 | 3170 | 2656 | 4438 | 2585 |
| IR 102818-10-266-3-2-2-6 | *DTY_1.1_* + *DTY_2.1_* | 115 | 2830 | 247 | 3480 | 1567 | -225 | 1515 |
| Samba Mahsuri* | *-* | 0 | 1124 | 15 | 3137 | 1590 | 3456 | 103 |
| Trial Mean | *-* | 156±73 | 3186±688 | 126±101 | 5824±1315 | 3131±1126 | 4389±1186 | 1751±375 |

*GY (Kg ha^-1^) for Samba Mahsuri, For PLs: GY advantage (Kg ha^-1^)

**Supplementary Table S3. Performance and allelic composition of pyramided lines with *DTY_2.2_* + *DTY_4.1_, DTY_2.2_*, and *DTY_4.1_* under NS and RS conditions in WS2015.**

|  |  | **WS 2015** | | | | | | **DS 2016** | |  | **%** | | | |
| --- | --- | --- | --- | --- | --- | --- | --- | --- | --- | --- | --- | --- | --- | --- |
| **PLs** | **Designation** | **DTF_NS** | **DTF_RS** | **PHT_NS** | **PHT_RS** | **GY_NS** | **GY_RS** | **GY_NS** | **GY_RS** | **QTL** | **A** | **B** | **H** | **R** |
| **6** | IR 99734:1-33-304-1-5-8 | 83 | 88 | 108 | 80 | 5404 | 175 | 4400 | 340 | *DTY_2.2_* + *DTY_4.1_* | 56.5 | 29.8 | 12.7 | 1 |
| **47** | IR 99734:1-33-304-1-5-10 | 81 | 81 | 109 | 71 | 4880 | 190 | 4107 | 250 | *DTY_2.2_* + *DTY_4.1_* | 47 | 51.8 | 1.2 | 0 |
| **2** | IR 99734:1-33-69-1-12-4 | 81 | 82 | 102 | 80 | 4519 | 542 | 3697 | 188 | *DTY_2.2_* + *DTY_4.1_* | 48.2 | 48.8 | 3 | 0 |
| **5** | IR 99734:1-33-69-1-39-2 | 82 | 83 | 111 | 77 | 3997 | 317 | 3531 | 246 | *DTY_2.2_* + *DTY_4.1_* | 54.2 | 42.3 | 3.6 | 0 |
| **11** | IR 99734:1-33-69-1-22-6 | 88 | 95 | 99 | 73 | 4183 | 307 | 4581 | 97 | *DTY_2.2_* + *DTY_4.1_* | 44 | 53.6 | 2.4 | 0 |
| **12** | IR 99734:1-33-69-1-12-8 | 82 | 86 | 104 | 74 | 4047 | 278 | 4682 | 272 | *DTY_2.2_* + *DTY_4.1_* | 46.4 | 49.4 | 4.2 | 0 |
| **13** | IR 99734:1-33-69-1-22-7 | 88 | 102 | 105 | 72 | 4626 | 147 | - | - | *DTY_2.2_* + *DTY_4.1_* | 41.1 | 54.8 | 4.2 | 0 |
| **21** | IR 99734:1-33-69-1-20-10 | 85 | 94 | 103 | 75 | 4498 | 105 | - | - | *DTY_2.2_* + *DTY_4.1_* | 41.7 | 57.1 | 1.2 | 0 |
| **22** | IR 99734:1-33-69-1-25-4 | 83 | 85 | 105 | 73 | 4131 | 125 | - | - | *DTY_2.2_* + *DTY_4.1_* | 46.4 | 52.4 | 1.2 | 0 |
| **28** | IR 99734:1-33-69-1-22-2 | 89 | 97 | 110 | 72 | 4288 | 219 | 4159 | 168 | *DTY_2.2_* + *DTY_4.1_* | 47.6 | 48.8 | 3.6 | 0 |
| **29** | IR 99734:1-33-69-1-9-3 | 82 | 83 | 109 | 79 | 3909 | 434 | 3655 | 217 | *DTY_2.2_* + *DTY_4.1_* | 42.9 | 45.2 | 11.9 | 0 |
| **30** | IR 99734:1-33-69-1-25-5 | 83 | 88 | 103 | 72 | 4258 | 217 | 3786 | 266 | *DTY_2.2_* + *DTY_4.1_* | 47 | 47 | 6 | 0 |
| **34** | IR 99734:1-33-69-1-39-8 | 83 | 85 | 108 | 70 | 4518 | 154 | - | - | *DTY_4.1_* | 42.9 | 47.1 | 10 | 0 |
| **37** | IR 99734:1-33-69-1-39-6 | 81 | 84 | 108 | 85 | 4508 | 498 | 5154 | 296 | *DTY_2.2_* + *DTY_4.1_* | 54.2 | 39.3 | 6.5 | 0 |
| **40** | IR 99734:1-33-69-1-22-4 | 90 | 103 | 104 | 72 | 4262 | 98 | - | - | *DTY_2.2_* + *DTY_4.1_* | 42.9 | 53.6 | 3.6 | 0 |
| **41** | IR 99734:1-33-69-1-20-9 | 84 | 89 | 107 | 81 | 4038 | 119 | - | - | *DTY_2.2_* + *DTY_4.1_* | 40.5 | 58.9 | 0.6 | 0 |
| **55** | IR 99734:1-33-69-1-12-10 | 83 | 83 | 108 | 78 | 4438 | 186 | 5793 | 132 | *DTY_2.2_* + *DTY_4.1_* | 47 | 47.6 | 5.4 | 0 |
| **58** | IR 99734:1-33-69-1-22-5 | 86 | 98 | 104 | 67 | 4673 | 58 | - | - | *DTY_2.2_* + *DTY_4.1_* | 37.5 | 53.6 | 8.9 | 0 |
| **59** | IR 99734:1-33-69-1-12-9 | 81 | 79 | 106 | 85 | 4353 | 204 | 4297 | 219 | *DTY_2.2_* + *DTY_4.1_* | 48.8 | 45.8 | 5.4 | 0 |
| **60** | IR 99734:1-33-69-1-20-6 | 84 | 92 | 109 | 70 | 4236 | 33 | - | - | *DTY_2.2_* + *DTY_4.1_* | 47.6 | 45.8 | 6.5 | 0 |
| **1** | IR 99734:2-23-174-3-3-1 | 88 | 101 | 105 | 55 | 3105 | 7 | - | - | *DTY_2.2_* | 38.1 | 53.6 | 8.3 | 0 |
| **3** | IR 99734:1-33-317-1-6-4 | 91 | 95 | 103 | 59 | 4933 | 38 | - | - | *DTY_2.2_* + *DTY_4.1_* | 38.7 | 58.3 | 3 | 0 |
| **4** | IR 99734:1-33-304-1-5-6 | 87 | 118 | 108 | 71 | 5133 | 11 | - | - | *DTY_2.2_* + *DTY_4.1_* | 48.2 | 47 | 4.8 | 0 |
| **7** | IR 99734:1-33-69-1-9-2 | 84 | 98 | 103 | 69 | 3639 | 27 | - | - | *DTY_2.2_* + *DTY_4.1_* | 47.6 | 50.6 | 1.8 | 0 |
| **8** | IR 99734:2-23-124-3-11-10 | 89 | 104 | 108 | 65 | 3759 | 8 | - | - | *DTY_2.2_* + *DTY_4.1_* | 39.9 | 52.4 | 2.4 | 5.4 |
| **9** | IR 99734:1-39-47-2-20-4 | 92 | 121 | 101 | 64 | 5076 | 104 | - | - | *DTY_4.1_* | 27.4 | 63.7 | 8.9 | 0 |
| **10** | IR 99734:1-33-304-1-5-7 | 83 | 90 | 100 | 71 | 4969 | 25 | - | - | *DTY_2.2_* + *DTY_4.1_* | 47.6 | 39.9 | 12.5 | 0 |
| **14** | IR 99734:1-39-47-2-20-3 | 92 | 117 | 100 | 59 | 5117 | 260 | 6221 | 39 | *DTY_4.1_* | 26.8 | 68.5 | 4.8 | 0 |
| **15** | IR 99734:1-33-69-1-20-2 | 91 | 107 | 106 | 66 | 4158 | -11 | - | - | *DTY_2.2_* + *DTY_4.1_* | 32.7 | 53 | 14.3 | 0 |
| **16** | IR 99734:2-23-124-3-2-7 | 87 | 89 | 108 | 71 | 3851 | 3 | - | - | *DTY_2.2_* + *DTY_4.1_* | 33.9 | 54.8 | 10.7 | 0.6 |
| **17** | IR 99734:1-61-10-1-13-9 | 84 | 86 | 101 | 67 | 3763 | 90 | 4049 | 224 | *DTY_2.2_* + *DTY_4.1_* | 52.4 | 44.6 | 3 | 0 |
| **18** | IR 99734:2-23-174-3-3-10 | 90 | 103 | 105 | 74 | 4171 | 74 | - | - | *DTY_2.2_* + *DTY_4.1_* | 44.6 | 40.5 | 14.3 | 0.6 |
| **19** | IR 99734:2-23-124-3-2-4 | 86 | 96 | 111 | 74 | 3785 | 8 | - | - | *DTY_2.2_* + *DTY_4.1_* | 36.3 | 46.4 | 17.3 | 0 |
| **20** | IR 99734:1-33-304-1-22-9 | 84 | 87 | 105 | 78 | 4289 | 98 | - | - | *DTY_2.2_* + *DTY_4.1_* | 44 | 40.5 | 15.5 | 0 |
| **23** | IR 99734:1-61-10-1-13-6 | 86 | 94 | 101 | 58 | 3243 | 96 | - | - | *DTY_2.2_* + *DTY_4.1_* | 43.5 | 37.5 | 19 | 0 |
| **24** | IR 99734:1-33-317-1-6-10 | 89 | 100 | 107 | 67 | 4602 | 70 | - | - | *DTY_2.2_* + *DTY_4.1_* | 45.8 | 52.4 | 1.8 | 0 |
| **25** | IR 99734:2-23-124-3-11-4 | 86 | 87 | 100 | 69 | 3266 | 131 | - | - | *DTY_2.2_* | 37.5 | 51.8 | 8.9 | 1.8 |
| **26** | IR 99734:1-61-10-1-13-5 | 91 | 103 | 98 | 61 | 3095 | 112 | - | - | *DTY_2.2_* + *DTY_4.1_* | 54.2 | 42.3 | 3.6 | 0 |
| **27** | IR 99734:2-23-124-3-2-10 | 86 | 87 | 112 | 76 | 3533 | 48 | - | - | *DTY_2.2_* + *DTY_4.1_* | 33.9 | 44.6 | 20.2 | 1.2 |
| **31** | IR 99734:1-39-47-2-20-1 | 93 | 114 | 97 | 67 | 4623 | 70 | - | - | *DTY_4.1_* | 34.5 | 60.1 | 5.4 | 0 |
| **32** | IR 99734:1-33-317-1-6-8 | 89 | 106 | 102 | 67 | 4890 | 67 | - | - | *DTY_2.2_* + *DTY_4.1_* | 42.9 | 54.8 | 2.4 | 0 |
| **33** | IR 99734:1-33-304-1-22-6 | 84 | 103 | 108 | 70 | 4075 | 12 | - | - | *DTY_2.2_* + *DTY_4.1_* | 50 | 44 | 6 | 0 |
| **35** | IR 99734:2-23-124-3-11-7 | 89 | 109 | 108 | 66 | 3424 | -3 | - | - | *DTY_2.2_* | 36.3 | 56 | 3 | 4.8 |
| **36** | IR 99734:1-33-304-1-22-8 | 88 | 113 | 106 | 77 | 4122 | -2 | - | - | *DTY_2.2_* + *DTY_4.1_* | 52.4 | 47 | 0.6 | 0 |
| **38** | IR 99734:2-23-174-3-3-2 | 90 | 92 | 107 | 70 | 3492 | 38 | - | - | *DTY_2.2_* + *DTY_4.1_* | 38.7 | 37.5 | 22 | 1.8 |
| **39** | IR 99734:1-33-304-1-5-4 | 83 | 91 | 105 | 75 | 4240 | 38 | - | - | *DTY_2.2_* + *DTY_4.1_* | 44 | 36.3 | 19.6 | 0 |
| **42** | IR 99734:1-33-317-1-6-9 | 89 | 109 | 111 | 65 | 4882 | 53 | - | - | *DTY_2.2_* + *DTY_4.1_* | 39.9 | 52.4 | 7.7 | 0 |
| **43** | IR 99734:2-23-124-3-11-3 | 86 | 91 | 99 | 68 | 2789 | 67 | - | - | *DTY_2.2_* | 34.5 | 47.6 | 17.9 | 0 |
| **44** | IR 99734:2-23-124-3-2-6 | 86 | 99 | 107 | 73 | 2836 | 18 | - | - | *DTY_2.2_* + *DTY_4.1_* | 48.8 | 47.6 | 2.4 | 1.2 |
| **45** | IR 99734:1-61-10-1-13-10 | 89 | 110 | 100 | 69 | 3168 | 157 | - | - | *DTY_2.2_* + *DTY_4.1_* | 53.6 | 43.5 | 3 | 0 |
| **46** | IR 99734:2-23-174-3-3-11 | 87 | 105 | 107 | 71 | 4170 | 20 | - | - | *DTY_2.2_* | 42.3 | 51.8 | 6 | 0 |
| **48** | IR 99734:1-33-69-1-20-3 | 85 | 81 | 101 | 80 | 4392 | 119 | - | - | *DTY_2.2_* + *DTY_4.1_* | 31.5 | 36.3 | 30.4 | 1.8 |
| **49** | IR 99734:2-23-124-3-11-5 | 89 | 111 | 107 | 67 | 3313 | 1 | - | - | *DTY_2.2_* + *DTY_4.1_* | 44 | 50 | 0.6 | 5.4 |
| **50** | IR 99734:1-33-69-1-9-4 | 84 | 82 | 99 | 83 | 4164 | 42 | - | - | *DTY_2.2_* + *DTY_4.1_* | 47 | 50.6 | 2.4 | 0 |
| **51** | IR 99734:1-33-317-1-6-1 | 91 | 118 | 108 | 65 | 5063 | 44 | - | - | *DTY_2.2_* + *DTY_4.1_* | 36.3 | 61.3 | 2.4 | 0 |
| **52** | IR 99734:1-39-47-2-20-2 | 94 | 117 | 99 | 66 | 4383 | 128 | - | - | *DTY_4.1_* | 28.6 | 60.7 | 10.7 | 0 |
| **53** | IR 99734:1-33-304-1-22-10 | 83 | 82 | 105 | 70 | 4225 | 16 | - | - | *DTY_2.2_* + *DTY_4.1_* | 53 | 44 | 3 | 0 |
| **54** | IR 99734:1-61-10-1-13-3 | 94 | 117 | 107 | 61 | 3800 | 53 | - | - | *DTY_2.2_* + *DTY_4.1_* | 50.6 | 47 | 2.4 | 0 |
| **56** | IR 99734:1-33-317-1-6-3 | 90 | 112 | 111 | 66 | 5343 | 85 | - | - | *DTY_2.2_* + *DTY_4.1_* | 38.7 | 61.3 | 0 | 0 |
| **57** | IR 99734:2-23-124-3-11-1 | 89 | 97 | 105 | 75 | 3433 | 25 | - | - | *DTY_2.2_* + *DTY_4.1_* | 39.9 | 48.8 | 6 | 5.4 |
| **61** | IR 99734:1-33-304-1-22-4 | 87 | 109 | 107 | 63 | 4362 | 37 | - | - | *DTY_2.2_* + *DTY_4.1_* | 44.6 | 49.4 | 6 | 0 |
| **62** | IR 99734:1-33-304-1-5-1 | 85 | 112 | 109 | 68 | 4784 | 29 | - | - | *DTY_2.2_* + *DTY_4.1_* | 49.4 | 44 | 6.5 | 0 |
| **63** | IR 99734:1-39-47-2-20-5 | 93 | 112 | 98 | 64 | 4618 | 387 | 5064 | 28 | *DTY_4.1_* | 28.6 | 69 | 2.4 | 0 |
| **64** | IR 99734:2-23-174-3-3-3 | 90 | 96 | 105 | 68 | 2658 | 27 | - | - | *DTY_2.2_* + *DTY_4.1_* | 54.2 | 41.7 | 3.6 | 0.6 |
| **65** | IR 99734:1-33-69-1-20-5 | 85 | 92 | 106 | 72 | 3614 | 51 | - | - | *DTY_2.2_* + *DTY_4.1_* | 41.7 | 50 | 8.3 | 0 |
| **66** | Samba Mahsuri | 105 | 130 | 101 | 74 | 1944 | 48 | - | - | No QTL | - | 100 | - | - |
| **67** | IR87728-75-B-B | - | - | - | - | - | - | - | - | *DTY_2.2_* + *DTY_4.1_* | 100 | - | - | - |
| **68** | IR87707-445-B-B-B | 85 | 96 | 115 | 79 | 6273 | 192 | 5337 | 872 | *DTY_2.2_* + *DTY_4.1_* | 52.0 | 47.0 | 1.0 | 0 |
|  |  |  |  |  |  |  |  |  |  |  |  |  |  |  |
|  | Trial Mean | 87 | 98 | 105 | 71 | 4180 | 114 | - | - |  |  |  |  |  |
|  | SED | 1 | 7 | 4 | 6 | 569 | 126 | - | - |  |  |  |  |  |

A: donor allele, B: recipient allele, H: heterozygous allele, R: recombinant allele.

**Supplementary Table S4. Comparison of mean grain yield of each group class calculated under reproductive stage drought stress and non stress conditions.**

|  | **WS 2015** | | **DS 2016** | |
| --- | --- | --- | --- | --- |
|  | GY_NS | GY_RS | GY_NS | GY_RS |
| **Group 1** | 4272 | 217 | 4308 | 223 |
| **Group 2** | 3408 | 34 | - | - |
| **Group 3** | 4351 | 83 | 5643 | 33 |
| **Group 4** | 4467 | 30 | - | - |
| **Group 5** | 5142 | 183 | 4254 | 295 |
| **Group 6** | 3414 | 101 | 4049* | 224* |

**mean of two lines only*

**Supplementary Table S5.** Comparison of mean grain yield of PLs with and without conserved allelic region on Chromosome 7 under NS and severe reproductive stage drought stress.

|  | **WS 2015_NS** | **DS 2016_NS** | **WS 2015_RS** | **DS 2016_RS** |
| --- | --- | --- | --- | --- |
| **PLs with conserved allelic region** | 4380 | 4296 | 250 | 236 |
| **PLs without conserved allelic region** | 4084 | 4979* | 69 | 33 |
| **Samba Mahsuri** | 4044 | 4951 | 48 | 0 |

*mean grain yield of four PLs, based on plant type and grain type other lines were not selected in WS 2015 to screen in DS 2016

NS: Non stress, RS: Reproductive stage drought stress

**Supplementary Table S6.** QTL interactions detected for grain yield under drought stress in marker assisted backcross experiment.

| ***QTL_i_*** | ***Interval_i_*** | ***Position_i_*** | ***QTL_j_*** | ***Interval_j_*** | ***Position_j_*** | **AA** | **h^2^(aa)** | **P** |
| --- | --- | --- | --- | --- | --- | --- | --- | --- |
| RM518-RM16368 | 6.7-8.9 | 7.9 | RM10-RM320 | 59.1-76.5 | 63.5 | 18.7 | 1.4 | ** |
| RM10-RM320 | 59.1-76.5 | 63.5 | RM296-RM566 | 43.1-49.6 | 46.6 | 33.5 | 6.8 | **** |

QTLi and QTLj: testing points i and j, respectively

Intervali and Intervali: the intervals of testing points i and j

AA additive-by-additive effect: depicted as % of trial mean

h^2^ (aa) heritability of additive-by-additive effect

**, **** Significant at P 0.01 and 0.0001 levels, respectively


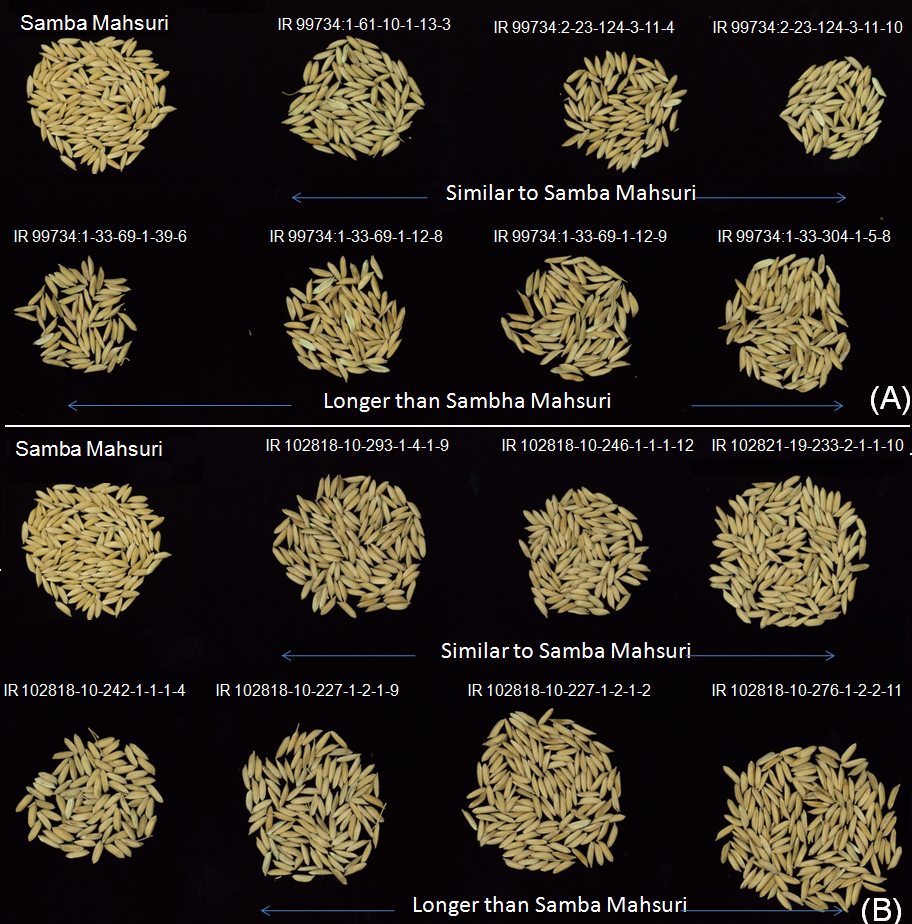


**Supplementary Fig. S1.** Variation in grain type of PLs compared to Samba Mahsuri (A) MAS (B) MARS in WS 2014.


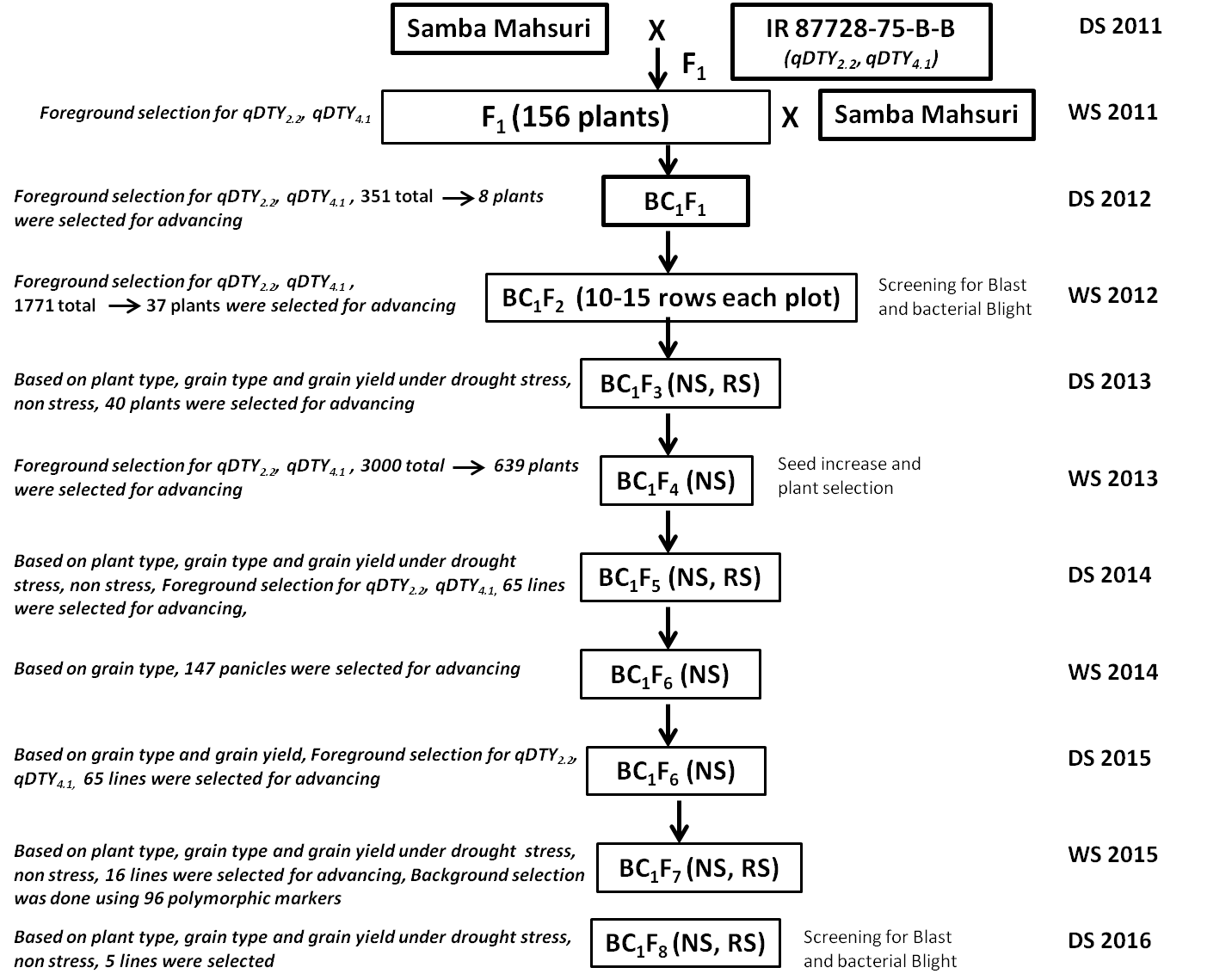


**Supplementary Fig. S2.** Scheme for the development of Samba Mahsuri pyramided lines and number of plants selected in every generation using marker assisted breeding approach.

*Plant type, grain type selection involves plants morphologically similar to recipient parent Samba Mahsuri

**
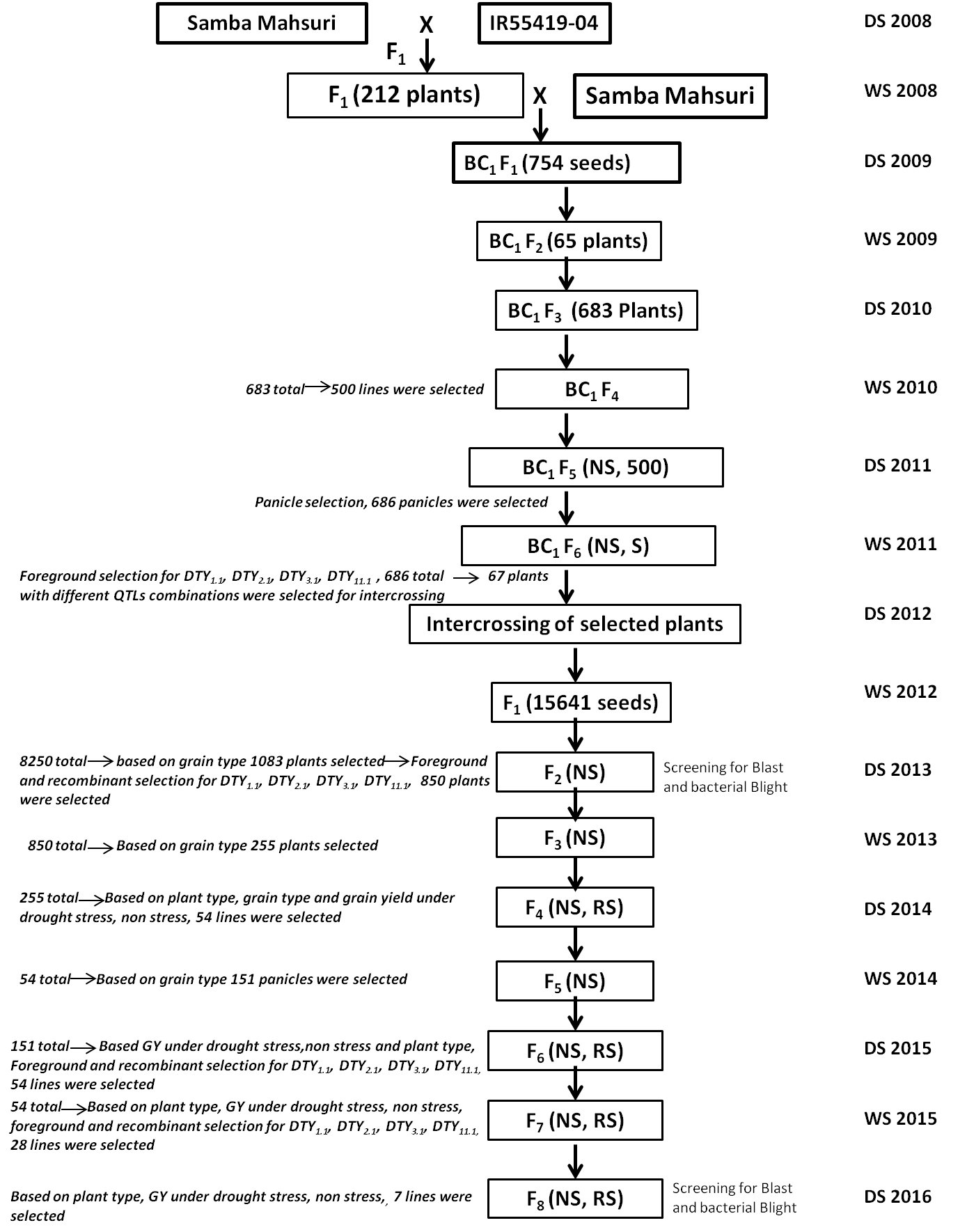
**

**Supplementary Fig. S3.** Scheme for the development of Samba Mahsuri pyramided lines and number of plants selected in every generation using partial marker assisted recurrent selection approach.

*Plant type, grain type selection involves plants morphologically similar to recipient parent Samba Mahsuri


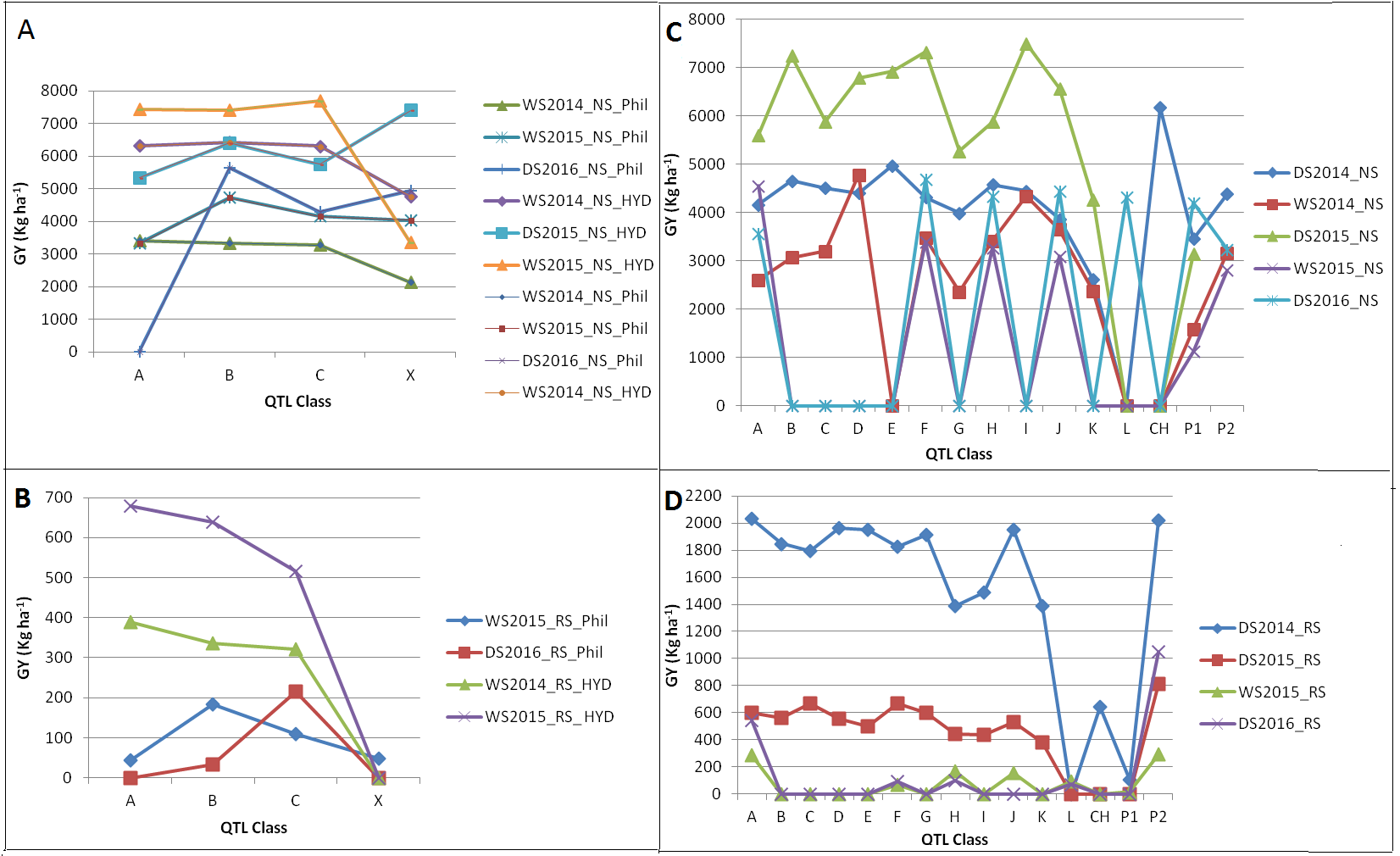


**Supplementary Fig. S4.** (A) Graph representing the QTL classes (X axis) and mean grain yield (Y axis) of MAS pyramided lines under NS (control) (B) Graph representing the QTL classes (X axis) and mean grain yield (Y axis) of MAS pyramided lines under under RS (C) Graph representing the QTL classes (X axis) and mean grain yield (Y axis) of MARS pyramided lines under NS (control) (D) Graph representing the QTL classes (X axis) and mean grain yield (Y axis) of MARS pyramided lines under RS.

*Supplementary Fig. S4 (A, B): QTL class A: DTY_2.2_, B: DTY_4.1_, C: DTY_2.2_ + DTY_4.1_, X: recipient parent (Samba Mahsuri); Supplementary Figure S6 (C, D): QTL class A: qDTY_1.1_ + qDTY_2.1_ + qDTY_3.1_ + qDTY_11.1_ + BR, B: qDTY_1.1_ + qDTY_2.1_ + qDTY_3.1_ + qDTY_11.1_, C: qDTY_1.1_ + qDTY_2.1_ + qDTY_3.1_, D: qDTY_1.1_ + qDTY_2.1_ + qDTY_3.1_ + BR, E: qDTY_1.1_ + qDTY_2.1_ + qDTY_11.1_, F: qDTY_1.1_ + qDTY_2.1_ + qDTY_11.1_ + BR, G: qDTY_2.1_ + qDTY_3.1_ + qDTY_11.1_, H: qDTY_1.1_ + qDTY_2.1_ + BR, I: qDTY_1.1_ + qDTY_3.1_ + BR, J: qDTY_1.1_ + qDTY_11.1_ + BR, K: qDTY_2.1_ + qDTY_11.1_ + BR, L: qDTY_1.1_ + BR, CH: Swarna, P1: Samba Mahsuri , P2: IR55419-04*.


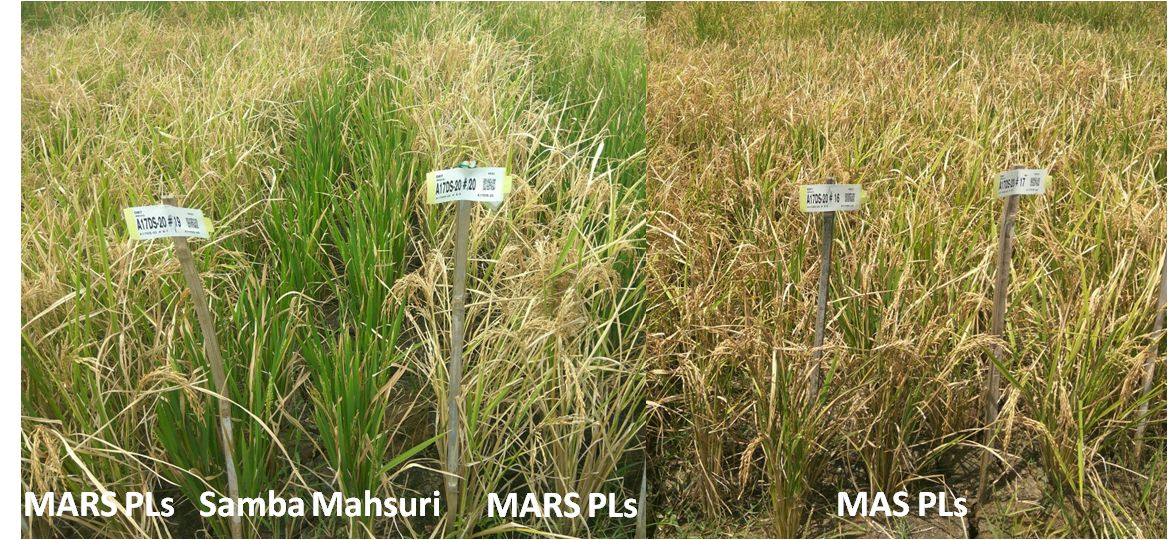


**Supplementary Fig. S5.** Grain yield advantage of MAS and MARS PLs over recipient parent under drought stress

**
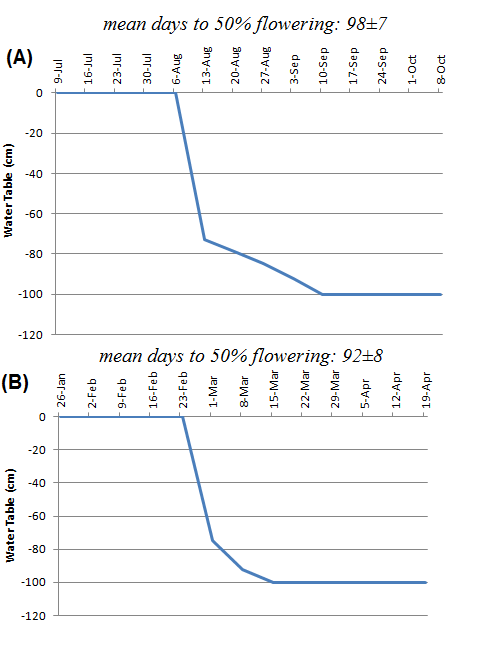
**

**Supplementary Fig. S6.** Soil water potential measured by parching water table level in (A) WS 2015 (mean days to 50% flowering of experiment: 98±7) (B) DS 2016 using polyvinyl chloride (PVC) pipe (mean days to 50% flowering of experiment: 92±8).
